# Cross-sectional and longitudinal associations between glycaemic measures and grip strength in people without diabetes in the UK Biobank cohort study

**DOI:** 10.1007/s41999-024-01119-2

**Published:** 2024-11-29

**Authors:** Antoneta Granic, Rachel Cooper, Christopher Hurst, Susan J. Hillman, Richard M. Dodds, Miles D. Witham, Avan A. Sayer

**Affiliations:** 1 AGE Research Group, Translational and Clinical Research Institute, Faculty of Medical Sciences, Newcastle University, The Health Innovation Neighbourhood, Biomedical Research Building, Newcastle Upon Tyne, NE4 5PL UK; 2 NIHR Newcastle Biomedical Research Centre, Newcastle Upon Tyne Hospitals NHS Foundation Trust, Cumbria Northumberland Tyne and Wear NHS Foundation Trust and Faculty of Medical Sciences Newcastle University, Newcastle Upon Tyne, UK

**Keywords:** Cohort study, Glycaemic measures, Grip strength, Probable sarcopenia

## Abstract

**Purpose:**

To investigate associations between glycaemic measures (HbA1c, random glucose), and grip strength (GS) in adults without prevalent diabetes.

**Methods:**

We included 381,715 UK Biobank participants aged 38–73 years without diabetes (any type) with complete baseline measures for GS and HbA1c (main analyses), and glucose (supplementary analyses). Cross-sectional sex- and age-stratified associations between each glycaemic measure, GS, and probable sarcopenia (low GS) were examined with regression analyses. Changes in GS over 8.9 years were classified into four groups (decline, stable low, stable high, or reference (increase or maintained within the normal range)) in 36,228 participants and associations with baseline glycaemic measures explored using multinomial regression.

**Results:**

Higher HbA1c (mmol/mol) was associated with weaker mean GS (kg) (regression coefficient and 95% confidence intervals (CI): − 0.08 (− 0.09, − 0.07)), and increased odds of probable sarcopenia (odds ratio (OR) and 95% CIs: 1.02 (95% CI: 1.01, 1.02)) in males and across the age groups. In females, higher HbA1c was associated with weaker mean GS only in mid-life (e.g., 50–59 years: − 0.06 (− 0.07, − 0.05)). In males, but not in females with repeated GS, higher HbA1c was associated with decreased odds of stable high (0.97 (0.96, 0.99) and increased odds of stable low (1.03 (1.01, 1.04)) GS pattern (0.98 (0.97, 0.980)) over the follow-up. The results for glucose in supplementary analyses were mixed, especially in females.

**Conclusions:**

The associations between HbA1c and GS in people without diabetes warrant replication and consideration of the effect on muscle strength when interventions to promote normoglycaemia are trialled.

**Supplementary Information:**

The online version contains supplementary material available at 10.1007/s41999-024-01119-2.

## Key summary points

**Aim:**

To investigate cross-sectional and longitudinal associations between glycaemic measures (HbA1c and random glucose) and grip strength (GS) in middle-aged and older adults without prevalent diabetes.

**Findings:**

A unit increase in HbA1c was associated with 1–3% higher odds of probable sarcopenia in males across all age groups, and 1–2% higher odds in middle-aged females at baseline. Over 9-year follow-up, males, but not females, with higher baseline HbA1c had decreased odds of having stable high GS pattern, and increased odds of having stable low GS pattern compared with reference (increase or maintained GS within the normal range).

**Message:**

Higher HbA1c may be associated with weaker GS in individuals without prevalent diabetes, but the findings warrant replication of the effect on muscle strength when interventions to promote normoglycaemia are trialled.

**Supplementary Information:**

The online version contains supplementary material available at 10.1007/s41999-024-01119-2.

## Introduction

Skeletal muscle is one of the most dynamic and plastic tissues in the human body contributing to multiple body functions. These functions include force and movement generation as well as metabolic functions such as protein and carbohydrate (glucose) storage and energy production [1]. Changes in skeletal muscle structure and function are associated with a decline in muscle strength and mass [1, 2] that is recognised as probable or definite sarcopenia when critical thresholds are crossed [3]. Sarcopenia is linked to impaired mobility, loss of independence and reduced quality and length of life [4].

At the cellular level, several age-related changes and mechanisms have been implicated in driving muscle ageing and sarcopenia, including mitochondrial dysfunction, inflammation, and dysregulation of nutrient-sensing mechanisms, such as those related to the muscle protein synthesis-breakdown cycle, fat infiltration, glucose dyshomeostasis and insulin resistance [5, 6]. For instance, skeletal muscle is responsible for 80% of postprandial glucose uptake and is central to energy balance within myofibers and throughout the body [6]. With ageing, there is decreased ability of insulin to stimulate muscle to clear glucose from the circulation leading to insulin resistance of skeletal muscle. Insulin resistance in skeletal muscle is viewed as the initiating or primary defect in type 2 diabetes [7].

Low skeletal muscle strength (assessed by, for example, grip strength, GS) is regarded as a biomarker of ageing and mortality [8] and is a key element of sarcopenia diagnosis [3]. Epidemiological studies suggest a bi-directional relationship between muscle strength (GS) and diabetes [9–16], with some inconsistencies also being reported [17, 18]. Middle-aged and older adults with diabetes have lower muscle strength (GS) [9–11], accelerated loss of muscle mass and function [12], and a higher prevalence of sarcopenia [13]. Conversely, low GS was observed as a risk factor for incident diabetes in healthy adults and in those with prediabetes (hyperglycaemia or dysglycaemia assessed by glycaemic measures such as HbA1c, insulin, plasma glucose levels, or glucose tolerance test) [9, 14–16].

Whether glycaemic measures relate to muscle strength and influence muscle strength change in middle-aged and older adults without diagnoses of diabetes or hyperglycaemia has been less often explored [10, 18–20]. Studies have reported inconsistent results and there is some evidence of a sex-specific pattern with significant associations being more often reported in males than in females [21, 22].

Taken together, this suggests that dysglycaemia preceding diabetes may be a risk factor for loss of muscle function and probable sarcopenia, possibly starting in mid-adulthood. To test this hypothesis, the current study used cross-sectional and longitudinal data from the UK Biobank cohort to investigate sex- and age-stratified associations between baseline glycaemic measures (HbA1c (main analyses) and random glucose (supplementary analyses)) and GS and GS change over 9 years in middle-aged and older adults without prevalent diabetes.

## Methods

### Study population and design

UK Biobank is a cohort study of over 500,000 participants aged 37–73 years designed to investigate genetic and lifestyle determinants of common diseases of middle and old age. Participants were recruited for baseline health questionnaire, functioning assessments, and biological samples collection at 22 centres across England, Wales, and Scotland between 2006–2010, as detailed elsewhere [23] and in the Supplementary Information, Appendix 1. A sub-set of > 100,000 participants were re-invited from 2014 onwards to selected assessment centres for brain, heart, and body imaging visits and to repeated baseline measures (i.e., the imaging visit) [24].

### Analytic sample

We used Eastwood et al.’s [25] algorithm for diabetes ascertainment to classify participants at baseline as ‘diabetes likely’ based on self-reported diabetes diagnosis and diabetes medication (insulin, sulfonylureas, glitazones, meglitinides, or acarbose) or ‘diabetes unlikely’ (i.e. participants likely to be free of diabetes). Information from both touchscreen questionnaire and an interview with a research nurse at an Assessment Centre was used to identify the ‘diabetes unlikely’ sample who were the focus of our analyses (detailed in Supplementary Information, Tables S1 and S2). Briefly, we classified 470,409 participants as ‘diabetes unlikely’ (thereafter ‘without prevalent diabetes’). Of those, 469,904 had data for HbA1c, glucose, and GS after removing extreme values for all three measures (n = 505; detailed in Supplementary Information, Appendix 1, Methods). This left 381,715 participants with complete measures for HbA1c, glucose, and GS without prevalent diabetes at baseline (i.e., analytic sample). Of those, 36,228 had GS measured at the imaging visit approximately 8.9 years later (Supplementary Information, Figure S1).

### Glycaemic measures

Blood was collected at baseline and the concentration of HbA1c (mmol/mol) was measured using Bio-Rad II Turbo analysers (Bio-Rad Laboratories, Inc.) and a HPLC method at baseline [26]. Random (non-fasting) glucose (mmol/l) was measured using Beckman Coulter AU5800 analyser (Beckman Coulter Ltd., UK) via an enzymatic method. Both measures were analysed as continuous variables in all participants, males, and females.

All biochemical analyses were performed in a central laboratory, and laboratory variables adjusted by UK Biobank before biomarker data release as detailed in the UK Biobank Biomarker protocol [26].

### Grip strength and probable sarcopenia at baseline

Grip strength (GS) was measured in each hand using a Jamar J00105 hydraulic handheld dynamometer as detailed in Supplementary Information, Appendix 1. Maximum GS (continuous, kg) was used in subsequent analyses and categorised as < 27 kg in males and < 16 kg in females to identify probable sarcopenia (low GS) based on the European Working Group on Sarcopenia in Older People 2 (EWGSOP2) algorithm [3].

### Grip strength patterns of change over 9 years

As GS was only assessed at two time points (at baseline and follow-up on average 8.9 years later), we used patterns of change to minimise the effects of regression to the mean and measurement error as previously described in [27, 28] and detailed in Supplementary Information, Appendix 1. Briefly, age (per decade) and sex-specific Z-scores of GS at baseline and imaging visits (range: 3.8–12.8 years) were grouped into three categories: low (< − 1), intermediate (between − 1 and 1), and high (> 1). These Z-score groups were used to define four sex- and age-specific (per decade) patterns of GS change: (a) low/intermediate at baseline and intermediate/high at the imaging visit (reference; thereafter ‘increase or maintained within the normal range’); (b) high at baseline and low/intermediate at imaging visit, or intermediate at baseline and low at follow-up (decline); (c) high at both visits (stable high), and (d) low at both visits (stable low) [27, 28].

### Potential covariates

Covariates are described in detail in Supplementary Information, Appendix 1 and presented in Table 1. Briefly, for all analyses we included the following baseline factors: (a) sociodemographic (age, sex, the Townsend Deprivation Index, education); (b) health-related (the number of body systems affected by at least one long-term conditions (LTC) as in [28]), and (c) lifestyle factors (BMI (weight/height^2^, kg/m^2^), smoking, alcohol intake status, self-reported leisure time physical activity (LTPA) based on the metabolic equivalent of task (MET) as in [29], and fruit and vegetables intake (g/day) as in [30]).

**Table 1:** Descriptive characteristics of the analytic sample without prevalent diabetes at baseline in UK Biobank (n = 381,715)

Characteristic	Males	Females
n (%)	172,795 (45.3)	208,920 (54.7)
Age at baseline, years, mean (SD)	56.5 (8.2)	56.3 (8.0)
Age group, years		
< 50	41,747 (24.2)	49,776 (23.8)
50–59	55,559 (32.2)	72,035 (34.5)
60–64	41,219 (23.8)	50,589 (24.2)
≥ 65	34,270 (19.8)	36,520 (17.5)
Time between visit, years, mean (SD)	8.9 (1.8)	8.9 (1.7)
Maximum GS at baseline, kg, mean (SD)	42.0 (9.0)	25.1 (6.4)
Probable sarcopenia^a^ at baseline, n (%)	6846 (4.0)	11,984 (5.7)
Maximum GS at imaging visit, kg, mean (SD)	40.0 (8.6)	24.6 (6.0)
Max GS pattern of change, n (%)		
Reference^b^	12,248 (70.2)	13,604 (72.5)
Decline	2777 (15.9)	2987 (15.9)
Stable high	1262 (7.2)	1064 (5.7)
Stable low	1164 (6.7)	1122 (6.0)
HbA1c, mmol/mol, mean (SD)	35.1 (3.9)	35.1 (3.9)
Random glucose, mmol/l, mean (SD)	5.0 (0.7)	5.0 (0.7)
Education, years, n (%)		
7	28,550 (16.7)	34,515 (16.7)
10	41,803 (24.4)	60,381 (29.2)
13	17,964 (10.5)	25,042 (12.1)
15	7614 (4.5)	12,055 (5.8)
19	15,546 (9.1)	9231 (4.5)
20 (reference)	59,544 (34.8)	65,662 (31.7)
Townsend deprivation index in fifths, n (%)		
Q1 (least deprived)	35,600 (20.6)	42,462 (20.4)
Q2	35,085 (20.3)	42,236 (20.3)
Q3	34,422 (20.0)	42,606 (20.4)
Q4	33,892 (19.7)	42,286 (20.3)
Q5 (most deprived) (reference)	33,485 (19.4)	38,975 (18.7)
Long-term conditions (excluding diabetes), n (%)		
0	46,547 (26.9)	55,346 (26.5)
1	60,704 (35.1)	67,369 (32.2)
2	39,531 (22.9)	46,475 (22.2)
≥ 3 (reference)	26,011 (15.1.)	39,724 (19.0)
BMI, kg/m^2^, mean (SD)	27.6 (4.0)	26.9 (5.0)
BMI categories, kg/m^2^, *N* (%)		
Normal (< 25)	45,239 (26.3)	85,191 (40.9)
Overweight (25–30)	86,880 (50.4)	77,374 (37.1)
Obese (> 30)	40,216 (23.3)	45,961 (22.0)
Smoking status, n (%)		
Never (reference)	85,684 (49.8)	123,947 (59.5)
Previous	64,955 (37.7)	65,591 (31.5)
Current	21,530 (12.5)	18,639 (9.0)
Alcohol intake status, n (%)		
Never	4295 (2.5)	11,217 (5.4)
Previous	5626 (3.3)	7227 (3.5)
Current	162,689 (94.3)	190,259 (91.2)
Leisure-time PA (MET-min/week), n (%)		
Low (< 600)	63,469 (39.1)	95,690 (49.7)
Moderate (600–3000)	81,521 (50.2)	87,388 (45.4)
High (≥ 3000) (reference)	17,399 (10.7)	9496 (4.9)
Fruit and vegetable intake^c^, g/day, n (%)		
< 400	109,965 (63.8)	106,791 (51.1)
≥ 400 (reference)	62,516 (36.2)	101,990 (48.9)

GS, grip strength; MET, metabolic equivalent of task; PA, physical activity; SD, standard deviation

^a^Low GS: < 27 kg (males) and < 16 kg (females); ^b^increase or maintained within the normal range. ^c^corresponding to < 5 and ≥ 5 a day

All covariates had < 5% of missingness except LTPA (e.g., BMI 0.3%, alcohol 0.1%, and LTPA 6% in males; Supplementary Information, Table S3) and were imputed with mean (for continuous variable) or reference group (for categorical variable) for multivariable analyses.

### Statistical analyses

Descriptive statistics (frequencies, percentages, and means with standard deviations (SD)) were used to describe males and females in the analytic sample at baseline (Table 1). All multivariable analyses were split into main (for HbA1c) and supplementary (for random glucose) analyses, and the latter reported in Supplementary Information, Appendix 1 and Appendix 2 as detailed below. Specifically, linear and logistic regression models as appropriate were used to examine cross-sectional associations between glycaemic measures and GS separately by sex and age group (Tables 2 and 3 for HbA1c and Supplementary Information, Appendix 2 for glucose). For linear regression, two models were fitted to investigate the linear associations between HbA1c, glucose and GS at baseline after testing the linearity assumptions (detailed in Supplementary Information, Appendix 1 and Table S4). Descriptive statistics (means (SD)) for GS and glycaemic measures were examined across the age group in both sexes (Table S5). Model 0 was unadjusted, and Model 1 was fully adjusted for sociodemographic, health, and lifestyle factors. The same models were fitted for logistic regression analyses with probable sarcopenia (low GS) as an outcome in males and females by age groups.

**Table 2:** Associations between HbA1c and grip strength in participants without prevalent diabetes at baseline in UK Biobank (n = 381,715)

Males				Females			
All (n = 172,795)				All (n = 208,920)			
Model	Regression coefficient^a^	95% CI	*p *value	Model	Regression coefficient^a^	95% CI	*p *value
HbA1c (mmol/mol)							
M0	− 0.20	− 0.21, − 0.19	< 0.001	M0	− 0.21	− 0.22, − 0.21	< 0.001
M1	− 0.08	− 0.09, − 0.07	< 0.001	M1	− 0.02	− 0.03, − 0.02	< 0.001
Age group				Age group			
< 50 years				< 50 years			
M0	− 0.11	− 0.13, − 0.09	< 0.001	M0	− 0.09	− 0.11, − 0.08	< 0.001
M1	− 0.12	− 0.14, − 0.10	< 0.001	M1	− 0.07	− 0.08, − 0.05	< 0.001
50–59 years				50–59 years			
M0	− 0.09	− 0.11, − 0.07	< 0.001	M0	− 0.09	− 0.1, − 0.08	< 0.001
M1	− 0.09	− 0.11, − 0.07	< 0.001	M1	− 0.06	− 0.07, − 0.05	< 0.001
60–64 years				60–64 years			
M0	− 0.09	− 0.11, − 0.07	< 0.001	M0	− 0.01	− 0.03, 0.001	0.06
M1	− 0.09	− 0.11, − 0.07	< 0.001	M1	0.01	− 0.01, − 0.02	0.3
≥ 65 years				≥ 65 years			
M0	− 0.07	− 0.09, − 0.05	< 0.001	M0	− 0.01	− 0.03, 0.005	0.17
M1	− 0.06	− 0.08, − 0.04	< 0.001	M1	− 0.01	− 0.002, 0.01	0.79

GS, grip strength. ^a^Regression coefficients represent the difference in mean GS (in kg) per 1 unit change in glycaemic measure

Model 0 (M0) is unadjusted

Model 1 (M1) is adjusted for age (in all), years of education, Townsend index of deprivation, number of long-term conditions, and BMI, leisure-time physical activity, smoking status, alcohol drinking status, and fruit and vegetable intake

**Table 3:** Odds ratios and 95% CIs of the associations between HbA1c and grip strength in participants without prevalent diabetes at baseline in UK Biobank (n = 381,715)

Males			Females		
Model	OR (95% CI)	*p *value	Model	OR (95% CI)	*p *value
HbA1c (mmol/mol)					
M0	1.04 (1.04, 1.05)	< 0.001	M0	1.06 (1.05, 1.06)	< 0.001
M1	1.02 (1.01, 1.02)	< 0.001	M1	1.01 (1.00, 1.01)	0.04
Age group			Age group		
< 50 years			< 50 years		
M0	1.05 (1.03, 1.06)	< 0.001	M0	1.06 (1.05, 1.08)	< 0.001
M1	1.03 (1.01, 1.04)	0.001	M1	1.02 (1.002, 1.03)	0.03
50–59 years			50–59 years		
M0	1.04 (1.03, 1.05)	< 0.001	M0	1.04 (1.03, 1.05)	< 0.001
M1	1.02 (1.01, 1.03)	< 0.001	M1	1.01 (1.003, 1.02)	0.008
60–64 years			60–64 years		
M0	1.03 (1.02, 1.04)	< 0.001	M0	1.01 (1.002, 1.02)	0.02
M1	1.01 (1.001, 1.02)	0.03	M1	0.99 (0.98, 1.002)	0.13
≥ 65 years			≥ 65 years		
M0	1.02 (1.01, 1.03)	< 0.001	M0	1.02 (1.01, 1.03)	< 0.001
M1	1.01 (1.001, 1.02)	0.03	M1	1.01 (1.00, 1.02)	0.07

Model 0 is unadjusted

Model 1 adjusted for age (in all), years of education, Townsend index of deprivation, number of long-term conditions, BMI, leisure-time physical activity, smoking status, alcohol intake status, and fruit and vegetable intake

Adjusted multinomial regression models were used to examine the sex-specific associations between glycaemic measures at baseline and GS patterns of change over 8.9 years follow-up in all participants with two measures of GS (Table 4 (HbA1c) and Supplementary Information, Appendix 2 for glucose).

**Table 4:** Associations between HbA1c and patterns of grip strength change in participants without prevalent diabetes at baseline in UK Biobank (n = 36,228)

All			Male		Female	
n = 36,228			n = 17,451		n = 18,777	
GS pattern^a^	OR (95% CI)^b^	*p-*value	OR (95% CI)^b^	*p-*value	OR (95% CI)^b^	*p-*value
Decline	0.99 (0.98, 1.0)	0.003	1.00 (0.99, 1.01)	0.74	0.98 (0.97, 0.99)	< 0.001
Stable high	0.98 (0.97, 0.98)	< 0.001	0.97 (0.96, 0.99)	< 0.001	0.99 (0.97, 1.00)	0.13
Stable low	1.02 (1.01, 1.03)	0.005	1.03 (1.01, 1.04)	0.001	1.01 (0.99, 1.02)	0.47

^a^GS (grip strength) pattern ‘increase or maintained within the normal range’ (reference). ^b^Multinominal regression models; OR (odds ratio) of belonging to a GS pattern per unit increase in HbA1c (mmol/mol)

Models are adjusted for years of education, Townsend index of deprivation, number of long-term conditions, and BMI, leisure-time physical activity, smoking status, alcohol drinking status, and fruit and vegetable intake

All statistics were conducted using IBM SPSS (V.29; IBM Corporation, Armonk, NY, USA).

### Supplementary and sensitivity analyses

Supplementary and several sensitivity analyses were performed and detailed in Supplementary Information, Appendix 1 and Appendix 2. These included: (a) testing the rationale for sex- and age-group-stratified analyses in linear regression models that included sex x glycaemic measure (centred) and age group x glycaemic measure interaction terms (Table S6); (b) linear, logistic, and multinomial regression models for the associations between random glucose and GS across the age groups in both sexes (Tables S7–S9); for both glycaemic measures (c) adjusting key multivariable models with height instead of BMI and by excluding LTC to test the robustness of the effects reported in the main analyses (Tables S10–S15); (d) repeating key multivariable analyses after excluding 2417 participants with HbA1c ≥ 48 mmol/mol (≥ 6.5%) and/or glucose ≥ 11.1 mmol/l to account for undiagnosed diabetes (Tables S16–S18), and (e) in the analytic sample with complete covariates (n = 350,217) to examine the robustness of single imputations for covariates with missing values (Tables S19–S21).

## Results

### Characteristics of participants in the analytic sample

The analytic sample for cross-sectional analyses consisted of 381,715 participants without prevalent diabetes (54.7% female; mean age 56.3 years (SD = 8.0)) at baseline, and for the analyses of change in grip strength 36,228 participants (51.8% female; mean age 54.5 years (SD = 7.4)) (Supplementary Information, Figure S1). Table 1 presents the descriptive characteristics by sex in the analytic sample at baseline.

### Cross-sectional associations between glycaemic measures and grip strength

#### Linear associations of HbA1c and grip strength at baseline

We observed dose–response relationships between glycaemic measures, GS, and age groups (< 50, 50–59, 60–64, ≥ 65 years) in which GS decreased and glycaemic measures increased with age (Supplementary Information, Table S5). Linear models fitted these data the best (Table S4). Table 2 reports regression coefficients (unstandardised) of the associations between HbA1c and GS in males and females, and by age groups in each sex. In adjusted model (M1), higher HbA1c was associated with weaker mean GS (kg) (regression coefficient and 95% confidence intervals (CI): − 0.08 (− 0.09, − 0.07)) in males and across the age groups (0.06 to 0.12 kg lower mean GS per unit increase in HbA1c).

In females, higher HbA1c was associated with weaker mean GS in middle-aged (< 50 and 50–59 years) but not older age groups (60–64 and ≥ 65 years), a 0.7 and 0.6 kg lower mean GS per unit increase in HbA1c, respectively (Table 2).

#### Probable sarcopenia

Table 3 shows the odds ratios (OR 95% CI) of probable sarcopenia (GS < 27 kg in males and < 16 kg in females) per unit increase in HbA1c for each sex at baseline from unadjusted (M0) and fully adjusted models (M1). In males, a 1 unit increase in HbA1c was associated with 1–3% (95% CI: 0.01, 0.04) higher odds of probable sarcopenia across the age groups. Lower ORs and less consistent results were observed in females. A 1 unit increase in HbA1c was associated with 1–2% higher odds of probable sarcopenia in middle-aged but not older age groups (60 + years) (Table 3).

### Longitudinal associations between HbA1c and grip strength in the analytic sample

#### Associations between baseline HbA1c and patterns of grip strength change over 9 years

Patterns of GS change over 8.9 years in 36,228 participants (51.8% female) were as follows: 5,769 (15.9%) declined, 2,287 (6.3%) were stable low, 2,327 (6.4%) were stable high, and 25,867 (71.4%) were in the reference group (increase or maintained within the normal range). Table 4 presents independent associations between baseline HbA1c (continuous) and GS patterns of change compared to reference over ~ 9 years in participants without prevalent diabetes at baseline. In all participants, a unit increase in HbA1c was associated with decreased odds of stable high pattern (OR 0.98, 95% CI (0.97, 0.99)) and increased odds stable low pattern (1.02 (1.01, 1.03)), this also being observed in males (0.97 (0.96, 0.99) and 1.03 (1.01, 1.04), respectively). Associations in females were largely non-significant, except for decline pattern of GS change for HbA1c showing decreased ORs (0.98 (0.97, 0.99)—opposite to those observed in males.

### Supplementary and sensitivity analyses

#### Supplementary multivariable models for random glucose

Table S7 (Appendix 2) reports regression coefficients (95% CI) of the associations of glucose and GS. Whilst significant effects were observed in men (− 0.19 (− 0.25, − 0.14)), and especially in middle-aged groups (i.e., 0.26 kg lower mean GS per unit increase in glucose in those aged < 50 and 50–59 years, respectively), the results were mixed in females across the age groups. Additionally, less consistent evidence of associations was observed for glucose and the odds of probable sarcopenia (Table S8). In males, unit increase in glucose was associated with 13–14% higher odds (95% CI: 1.05, 1.24) of low GS in middle aged but not older age groups. In females, statistically significant associations were observed only in those aged 50–59 years, a 9% (95% CI: 1.04, 1.13) increased odds of low GS per unit increase in glucose. Furthermore, no associations were observed for glucose and patters of change in GS over ~ 9 years in all participants and by sex, except for decreased ORs (95% CI) in females (0.93 (0.87, 0.99) for decline pattern compared to reference (Table S9).

#### Sensitivity multivariable models with selected covariates and sub-sample analyses for both glycaemic measures

Linear and logistic regression models (M1) were repeated adjusting for height instead of BMI, and without adjusting for LTC (Supplementary Information, Tables S10–S15). 2,417 participants (0.6%) with HbA1c ≥ 48 mmol/mol (≥ 6.5%) and/or glucose ≥ 11.1 mmol/l were excluded from the analytic sample to account for those with undiagnosed diabetes [31] and key regression models repeated (M1) (Tables S16–S18). Also, main findings were compared to those in the analytic sample with complete values for all covariates (n = 350,217) (Tables S19–S21).

Although slightly attenuated, the effects (regression coefficients and ORs) were largely comparable with a few exceptions noted. Specifically, in linear regression models with height as a covariate, the effects of glycaemic measure in males were smaller and no longer statistically significant for glucose across the age groups. In females, the coefficients for HbA1c were positive and statistically significant in older age groups, and, although smaller, comparable for other ages and for glucose. However, the results for the associations between glycaemic measures and patterns of GS change with height as a covariate were compatible. Similar results were obtained by excluding LTC from the models across the analyses, and in the sub-sample without participants with potentially undiagnosed diabetes, with a few exceptions observed in females (e.g., HbA1c not associated with increased odds of low GS in all females but comparable across the age groups and for glucose). Lastly, the effects in the sub-sample without missing data for covariates were either slightly higher or comparable except glucose no longer associated with increased odds of low GS in females aged 50–59 years.

## Discussion

Using baseline and 9-year follow-up data from the UK Biobank cohort we investigated sex- and age-stratified associations between glycaemic measures and grip strength (GS) as a measure of probable sarcopenia in participants aged 38–73 years without diabetes at baseline. Several key observations were found, including (a) associations between higher levels of HbA1c and weaker GS in males across the age groups and in middle age in females; (b) 1–3% higher odds of probable sarcopenia (low GS) per unit increase in HbA1c in males across the age groups and 1–2% higher odds of low GS in middle age in females; (c) associations between higher baseline HbA1c and GS change such as increased odds of having stable low pattern over 9-year follow-up, and (d) less clear associations between glucose and GS in males and mostly non-significant and opposite associations in females (supplementary analyses). Although modest, these associations were independent of potential covariates examined, and largely robust to exclusion of participants with suspected undiagnosed diabetes and missing data for covariates in sensitivity analyses. Notably, higher HbA1c was associated with higher odds of probable sarcopenia in both sexes at baseline.

Some of our observations were consistent with findings from previous studies. For example, muscle strength (measured by knee extensor strength) remained lower in ~ 940 participants aged 25–96 years from the Baltimore Longitudinal Study of Aging for the highest quartiles of HbA1c (≥ 43 mmol/mol or ≥ 6.1%; hyperglycaemia) over up to 7.5 years of follow-up [19]. Similarly, higher HbA1c was associated with stable low GS pattern over ~ 9 years in our study. Each standard deviation (SD = 16 mg/dl) higher fasting plasma glucose (FPG) was associated with lower GS in males (but not in females) from the Rancho Bernardo Study of over 1,600 older adults aged ≥ 65 over median of 7 years [21]. However, the rate of change in GS was not affected by the levels of FPG in either sex over time. Equally, in an English study of over 1300 older adults aged between 60 and 70 years, those with normal glucose tolerance test but high glucose levels had lower GS, and this was evident especially for males [10]. However, no associations were found between prediabetes (defined as HbA1c 38 mmol/mol or 5.6%) and GS trajectories over ~ 16 years from mid to late adulthood in the Medical Research Council National Survey of Health and Development [18]. Also, most [10, 32, 33] but not all cross-sectional studies [9, 34] found an association between glycaemic measures, diabetes status (including prediabetes) and muscle strength (GS).

Here we have shown some evidence in support of there being muscle strength vulnerability to dysglycaemia especially among middle-aged adults (< 50 years and 50–59 years age group). Specifically, 1 unit increases in HbA1c and glucose were associated with 0.12 kg and 0.27 kg lower mean GS, respectively in males aged < 50 after adjustment for a range of covariates, including BMI and lifestyle. Smaller effects for HbA1c in middle-aged groups and mixed results for glucose across the age groups were observed in females (i.e., negative associations in those aged 50–59 years and a positive association in 65 + year-olds, and no associations between glucose and GS in other age groups). Although the mean differences in grip strength per unit change in HbA1c observed in our analyses were statistically significant, the estimated effect sizes were modest; therefore, it is unclear whether these differences are clinically meaningful. As an indication of their likely clinical significance, we refer to previous analyses of UK Biobank that reported that a 5 kg decrease in grip strength was associated with Hazard ratios for all-cause mortality in males and females of 1.16 and 1.20, respectively [35]. The mean difference of 0.12 kg reported in our study would therefore equate to an elevation in rates of all-cause mortality of only 0.39% in males and 0.48% in females.

The sex-specific differences in glycaemic measures-muscle strength relationship in adults without diabetes observed in the present study were also reported by others [21, 22]. Possible explanations may relate to differences in (a) body composition [36]; (b) gene expression profiles in skeletal muscle (> 2800 sex-differentially expressed genes, including those associated with BMI (*NOB1*, *DPYSL4*) and body fat percentage (*HOXC5*) in genome-wide association study) [37]; (c) myofibres type and fibre cross-sectional areas [38], and (d) hormonal aetiologies [39] between the sexes. Also, the results suggest midlife may be a sensitive period to glucose dyshomeostasis during a time of progressive changes in skeletal muscle [2], with potentially long-lasting consequences in late life.

Several possible mechanisms of glucose effects on muscle strength and mass have been proposed [40]. Insulin resistance [41] may exert negative effects on glucose homeostasis and balance between muscle protein synthesis (MPS) and breakdown (MPB) pathways (autophagy and mitochondrial dysfunction) in the muscle [40]. The MPS-MPB imbalance may further exacerbate loss of muscle mass and strength with ageing. Consequently, a vicious cycle is created in which these losses lead to decreased muscle surface area for glucose transport and storage, along with the inability of muscle to respond to insulin released by pancreas, further worsening glucose dyshomeostasis and feeding the pathways which accelerate loss of muscle strength and mass (sarcopenia), at which point the cycle starts again [40]. The model implies that the cycle could be stopped or prevented by restoring muscle insulin uptake and glucose homeostasis and drugs currently used to treat type 2 diabetes may be of interest for the treatment and prevention of sarcopenia [41, 42].

Our study has several strengths. We used a previously published algorithm to classify those without prevalent diabetes [25] in a large cohort. We employed several age- and sex-stratified analyses to investigate the influence of glycaemic measures on GS independently of age and several other potentially important covariates. We have largely confirmed the robustness of the main findings in sensitivity analyses.

Our study has several limitations. Although we have used a more conservative approach to select participants with ‘diabetes unlikely’ (from self-reported diagnosis and diabetes-related medication at baseline), there remains a possibility of misclassification of cases and inclusion of those with undiagnosed diabetes. We also recognise that the observed effects in both sexes are very modest and may have resulted from uncontrolled confounding (especially in the analyses of GS change), bias (see study limitations below), and chance (i.e., multiple associations assessed in a large sample). Also, their clinical significance is unclear. The extent to which the findings can be generalised is also unclear given ‘a healthy volunteer’ bias and predominantly White population in UK Biobank [43]. We were limited to two glycaemic measures that were assessed at baseline, and any change in glycaemic status (or incident diabetes) and change in covariates over ~ 9 years was not controlled for in longitudinal analyses. The latter may have contributed to conflicting findings for GS change in men and women. Also, GS was assessed only twice, and GS change was defined by sex- and age-adjusted patterns to avoid the regression to the mean. The observational nature of the study provides limited insights into mechanisms of how glycaemic measures may convey the risk of low GS. There is a possibility of ‘reverse causality’ in explored associations and low GS being a harbinger of dysglycemia and prediabetes [12, 14, 16, 44] in this cohort.

In conclusion, our study demonstrated that higher HbA1c may be associated with weaker GS as a measure of probable sarcopenia over 9-year follow-up in individuals without prevalent diabetes. These findings warrant replication and consideration of the effect on muscle strength when interventions to promote normoglycemia are trialled.

## Supplementary Information

Below is the link to the electronic supplementary material.

Supplementary file1 (DOCX 463 KB)

## Data Availability

UK Biobank data were obtained under application number 27567. Analysis code is available upon request to the corresponding author and is not publicly available. UK Biobank data are available to researchers with an approved request (https://www.ukbiobank.ac.uk/register-apply/).

## References

[1] Frontera WR, Ochala J (2015) Skeletal muscle: a brief review of structure and function. Calcif Tissue Int 96:183–195. 10.1007/s00223-014-9915-y 25294644 10.1007/s00223-014-9915-y

[2] Dodds RM, Syddall HE, Cooper R et al (2014) Grip strength across the life course: normative data from twelve British studies. PLoS ONE 9:e113637. 10.1371/journal.pone.0113637 25474696 10.1371/journal.pone.0113637 PMC4256164

[3] Cruz-Jentoft AJ, Bahat G, Bauer J et al (2019) Sarcopenia: revised European consensus on definition and diagnosis. Age Ageing 48:16–31. 10.1093/ageing/afy169 30312372 10.1093/ageing/afy169 PMC6322506

[4] Beaudart C, Demonceau C, Reginster JY et al (2023) Sarcopenia and health-related quality of life: a systematic review and meta-analysis. J Cachexia Sarcopenia Muscle 14:1228–1243. 10.1002/jcsm.13243 37139947 10.1002/jcsm.13243 PMC10235892

[5] Granic A, Suetterlin K, Shavlakadze T et al (2023) Hallmarks of ageing in human skeletal muscle and implications for understanding the pathophysiology of sarcopenia in women and men. Clin Sci (Lond) 137:1721–1751. 10.1042/CS20230319 37986616 10.1042/CS20230319 PMC10665130

[6] Merz KE, Thurmond DC (2020) Role of skeletal muscle in insulin resistance and glucose uptake. Compr Physiol 10:785–809. 10.1002/cphy.c190029 32940941 10.1002/cphy.c190029 PMC8074531

[7] DeFronzo RA, Tripathy D (2009) Skeletal muscle insulin resistance is the primary defect in type 2 diabetes. Diabetes Care 32(Suppl 2):S157–S163. 10.2337/dc09-S302 19875544 10.2337/dc09-S302 PMC2811436

[8] Sayer AA, Kirkwood TBL (2015) Grip strength and mortality: a biomarker of ageing? Lancet 386:226–227. 10.1016/S0140-6736(14)62349-7 25982159 10.1016/S0140-6736(14)62349-7

[9] Åström MJ, von Bonsdorff MB, Salonen MK et al (2021) Glucose regulation and grip strength in adults: findings from the Helsinki Birth Cohort Study. Arch Gerontol Geriatr 94:104348. 10.1016/j.archger.2021.104348 33516079 10.1016/j.archger.2021.104348

[10] Sayer AA, Dennison EM, Syddall HE et al (2005) Type 2 diabetes, muscle strength, and impaired physical function: the tip of the iceberg? Diabetes Care 28:2541–2542. 10.2337/diacare.28.10.2541 16186295 10.2337/diacare.28.10.2541

[11] Mori H, Kuroda A, Yoshida S, Yasuda T et al (2021) High prevalence and clinical impact of dynapenia and sarcopenia in Japanese patients with type 1 and type 2 diabetes: findings from the impact of Diabetes Mellitus on Dynapenia study. J Diabetes Investig 12:1050–1059. 10.1111/jdi.13436 33063949 10.1111/jdi.13436 PMC8169345

[12] Leenders M, Verdijk LB, van der Hoeven L et al (2023) Patients with type 2 diabetes show a greater decline in muscle mass, muscle strength, and functional capacity with aging. J Am Med Dir Assoc 14:585–592. 10.1016/j.jamda.2013.02.006 10.1016/j.jamda.2013.02.006 23537893

[13] Feng L, Gao Q, Hu K et al (2022) Prevalence and risk factors of sarcopenia in patients with diabetes: a meta-analysis. J Clin Endocrinol Metab 107:1470–1483. 10.1210/clinem/dgab884 34904651 10.1210/clinem/dgab884

[14] Qiu S, Cai X, Yuan Y et al (2022) Muscle strength and prediabetes progression and regression in middle-aged and older adults: a prospective cohort study. J Cachexia Sarcopenia Muscle 13:909–918. 10.1002/jcsm.12905 35068089 10.1002/jcsm.12905 PMC8978008

[15] Boonpor J, Parra-Soto S, Petermann-Rocha F et al (2021) Associations between grip strength and incident type 2 diabetes: findings from the UK Biobank prospective cohort study. BMJ Open Diabetes Res Care 9:e001865. 10.1136/bmjdrc-2020-001865 34353878 10.1136/bmjdrc-2020-001865 PMC8344322

[16] Brown EC, Buchan DS, Madi SA et al (2020) Grip strength cut points for diabetes risk among apparently healthy U.S. adults. Am J Prev Med 58:757–765. 10.1016/j.amepre.2020.01.016 32273132 10.1016/j.amepre.2020.01.016

[17] Marques-Vidal P, Vollenweider P, Waeber G et al (2017) Grip strength is not associated with incident type 2 diabetes mellitus in healthy adults: the CoLaus study. Diabetes Res Clin Pract 132:144–148. 10.1016/j.diabres.2017.08.004 28863331 10.1016/j.diabres.2017.08.004

[18] Norris T, Johnson W, Cooper R et al (2023) Associations between diabetes status and grip strength trajectory sub-groups in adulthood: findings from over 16 years of follow-up in the MRC National Survey of Health and Development. BMC Geriatr 23:213. 10.1186/s12877-023-03871-9 37016329 10.1186/s12877-023-03871-9 PMC10074704

[19] Kalyani RR, Metter EJ, Egan J et al (2015) Hyperglycemia predicts persistently lower muscle strength with aging. Diabetes Care 38:82–90. 10.2337/dc14-1166 25392294 10.2337/dc14-1166 PMC4274779

[20] Barzilay JI, Cotsonis GA, Walston J et al (2009) Insulin resistance is associated with decreased quadriceps muscle strength in nondiabetic adults aged >or=70 years. Diabetes Care 32:736–738. 10.2337/dc08-1781 19171728 10.2337/dc08-1781 PMC2660450

[21] Kalyani RR, Kim C, Ferrucci L et al (2015) Sex differences in the association of fasting and postchallenge glucose levels with grip strength among older adults: the Rancho Bernardo Study. BMJ Open Diabetes Res Care 3:e000086. 10.1136/bmjdrc-2015-000086 25969742 10.1136/bmjdrc-2015-000086 PMC4419462

[22] Zheng J, Zhang L, Jiang M (2022) Lower handgrip strength levels probably precede triglyceride glucose index and associated with diabetes in men not in women. J Diabetes Investig 13:148–155. 10.1111/jdi.13626 34228900 10.1111/jdi.13626 PMC8756317

[23] Sudlow C, Gallacher J, Allen N et al (2015) UK Biobank: an open access resource for identifying the causes of a wide range of complex diseases of middle and old age. PLoS Med 12:e1001779. 10.1371/journal.pmed.1001779 25826379 10.1371/journal.pmed.1001779 PMC4380465

[24] Littlejohns TJ, Holliday J, Gibson LM et al (2020) The UK Biobank imaging enhancement of 100,000 participants: rationale, data collection, management and future directions. Nat Commun 11:2624. 10.1038/s41467-020-15948-9 32457287 10.1038/s41467-020-15948-9 PMC7250878

[25] Eastwood SV, Mathur R, Atkinson M et al (2016) Algorithms for the capture and adjudication of prevalent and incident diabetes in UK Biobank. PLoS ONE 11:e0162388. 10.1371/journal.pone.0162388 27631769 10.1371/journal.pone.0162388 PMC5025160

[26] Fry D, Almond R, Moffatt S, et al. UK Biobank Enhancement Project Companion Document to Accompany Serum Biomarker Data. 2019; p. 1–16. Available from: https://biobank.ndph.ox.ac.uk/showcase/showcase/docs/serum_biochemistry.pdf

[27] Cooper R, Muniz-Terrera G, Kuh D (2016) Associations of behavioural risk factors and health status with changes in physical capability over 10 years of follow-up: the MRC National Survey of Health and Development. BMJ Open 6:e009962. 10.1136/bmjopen-2015-009962 27091818 10.1136/bmjopen-2015-009962 PMC4838696

[28] Hurst C, Murray JC, Granic A et al (2021) Long-term conditions, multimorbidity, lifestyle factors and change in grip strength over 9 years of follow-up: findings from 44,315 UK Biobank participants. Age Ageing 50:2222–2229. 10.1093/ageing/afab195 34657960 10.1093/ageing/afab195 PMC8581389

[29] Chudasama YV, Khunti KK, Zaccardi F et al (2019) Physical activity, multimorbidity, and life expectancy: a UK Biobank longitudinal study. BMC Med 17:108. 10.1186/s12916-019-1339-0 31186007 10.1186/s12916-019-1339-0 PMC6560907

[30] Foster HME, Celis-Morales CA, Nicholl BI et al (2018) The effect of socioeconomic deprivation on the association between an extended measurement of unhealthy lifestyle factors and health outcomes: a prospective analysis of the UK Biobank cohort. Lancet Public Health 3:e576–e585. 10.1016/S2468-2667(18)30200-7 30467019 10.1016/S2468-2667(18)30200-7

[31] Florkowski C (2013) HbA1c as a diagnostic test for diabetes mellitus - reviewing the evidence. Clin Biochem Rev 34:75–83 24151343 PMC3799221

[32] Nishimoto K, Doi T, Tsutsumimoto K, Nakakubo S et al (2022) Relationship between diabetes status and sarcopenia in community-dwelling older adults. J Am Med Dir Assoc 23:1718.e7-1718.e12. 10.1016/j.jamda.2022.07.020 36055368 10.1016/j.jamda.2022.07.020

[33] Qiu S, Cai X, Zhou X et al (2023) Muscle quality in relation to prediabetes phenotypes: a population-based study with mediation analysis. J Clin Endocrinol Metab. 10.1210/clinem/dgad630 37878955 10.1210/clinem/dgad630

[34] Miranda H, Bentes C, Resende M et al (1992) Association between handgrip strength and body composition, physical fitness, and biomarkers in postmenopausal women with metabolic syndrome. Rev Assoc Med Bras 2022(68):323–328. 10.1590/1806-9282.20210673 10.1590/1806-9282.20210673 35442358

[35] Celis-Morales CA, Welsh P, Lyall DM et al (2018) Associations of grip strength with cardiovascular, respiratory, and cancer outcomes and all cause mortality: prospective cohort study of half a million UK Biobank participants. BMJ 361:k1651. 10.1136/bmj.k1651 29739772 10.1136/bmj.k1651 PMC5939721

[36] Bredella MA (2017) Sex differences in body composition. Adv Exp Med Biol 1043:9–27. 10.1007/978-3-319-70178-3_2 29224088 10.1007/978-3-319-70178-3_2

[37] Oliva M, Muñoz-Aguirre M, Kim-Hellmuth S et al (2020) The impact of sex on gene expression across human tissues. Science 69:eaba3066. 10.1126/science.aba3066 10.1126/science.aba3066 PMC8136152 32913072

[38] Nuzzo JL (2024) Sex differences in skeletal muscle fiber types: a meta-analysis. Clin Anat 37:81–91. 10.1002/ca.24091 37424380 10.1002/ca.24091

[39] Sipilä S, Narici M, Kjaer M et al (2013) Sex hormones and skeletal muscle weakness. Biogerontology 14:231–245. 10.1007/s10522-013-9425-8 23636830 10.1007/s10522-013-9425-8

[40] Kalyani RR, Corriere M, Ferrucci L (2014) Age-related and disease-related muscle loss: the effect of diabetes, obesity, and other diseases. Lancet Diabetes Endocrinol 2:819–829. 10.1016/S2213-8587(14)70034-8 24731660 10.1016/S2213-8587(14)70034-8 PMC4156923

[41] Li M, Chi X, Wang Y et al (2020) Trends in insulin resistance: insights into mechanisms and therapeutic strategy. Signal Transduct Target Ther 7:216. 10.1038/s41392-022-01073-0 10.1038/s41392-022-01073-0 PMC9259665 35794109

[42] Witham MD, Granic A, Pearson E et al (2023) Repurposing drugs for diabetes mellitus as potential pharmacological treatments for sarcopenia - a narrative review. Drugs Aging 40:703–719. 10.1007/s40266-023-01042-4 37486575 10.1007/s40266-023-01042-4 PMC10371965

[43] Fry A, Littlejohns TJ, Sudlow C et al (2017) Comparison of sociodemographic and health-related characteristics of UK Biobank participants with those of the general population. Am J Epidemiol 186:1026–1034. 10.1093/aje/kwx246 28641372 10.1093/aje/kwx246 PMC5860371

[44] Hu S, Gu Y, Lu Z et al (2019) Relationship between grip strength and prediabetes in a large-scale adult population. Am J Prev Med 56:844–851. 10.1016/j.amepre.2019.01.013 31003803 10.1016/j.amepre.2019.01.013

