# Supplementary material for: Cross-sectional and longitudinal associations between glycaemic measures and grip strength in people without diabetes in the UK Biobank cohort study
Source: Eur Geriatr Med. 2024 Nov 29;16(1):67–77. doi: 10.1007/s41999-024-01119-2 (PMC11850503; doi:10.1007/s41999-024-01119-2)
Supplement: Supplementary file 1 — Supplementary file1 (DOCX 463 KB) [file 41999_2024_1119_MOESM1_ESM.docx]

**Supplementary Information**

**Appendix 1**

**Methods**

**Study population and UK Biobank recruitment process**

Nine million individuals aged 37 to 73 years living across the UK were invited to participate in the study between 2006 and 2010 [S1]. More than 502,000 were recruited (5.5% response rate) and invited to attend one of 22 assessment centres in England, Wales, and Scotland. The baseline visit included a self-completed touchscreen (TS) questionnaire, a computer assisted interview, an interview with a nurse at an Assessment Centre (AC), physical, functional, and anthropometric measurements, and blood, urine, and saliva collection [S2]. In all, the study collected detailed data about physical, mental, and lifestyle characteristics of participants with linkages to routinely collected clinical data via hospital inpatient and primary care records, death, and cancer registries. Starting from 2014, over 100,000 participants were re-invited to attend one of the four imaging centres across Central, North, South-East and South-West UK for brain, heart and body imaging, and follow-up assessments (i.e., the imaging visit/study). A centralised team was organised for training of the staff, quality assurance, and harmonization of data across the centres. Details about the imaging study are available at UK Biobank website (<https://www.ukbiobank.ac.uk/explore-your-participation/contribute-further/imaging-study>).

**Analytic sample**

We used the Prevalence algorithm 1 designed by Eastwood et al 2016 [25] to distinguish between diabetes presence / absence and to identify participants in whom ‘diabetes unlikely’ (described in S1 Appendix [25]) based on (a) absence of self-reported diabetes diagnosis (Data-field 2443 from touch-screen (TS) questionnaire + Data-field 20002 obtained at the Assessment Centre (AC) during an interview with a nurse), and (b) absence of diabetes medication (Data-field 20003 (47 medication/treatment codes, including insulin and metformin) + Data field 6177 (takes medication for cholesterol, blood pressure, and diabetes (insulin)) + Data-field 6153 (takes medication for cholesterol, blood pressure, diabetes (insulin), or takes exogenous hormones)). Data-fields 6177 and 6153 were used to extrapolate *insulin* variable. *Metformin* variable was extrapolated from Data-field 20003.

From these Data-fields, we derived three binary variables: *diabetes* (from 20002 Data-field (no = 0, yes = 1)), *diabetes1* (from 2443 Data-field (no = 0, yes = 1)), and *all diabetes drugs* (no = 0 (takes no diabetes drugs), yes = 1 (takes one or more diabetes drugs, including metformin and insulin)), and used the following combination codes (Table S1) to identify participants with ‘diabetes unlikely’ (i.e., no prevalent diabetes; Table S1).

Of 500,234 participants at baseline, 472,438 were classified as ‘diabetes unlikely’, 25,627 as ‘diabetes likely’, and 2,169 had missing data on at least one of the three diabetes variables described above. For more conservative approach and to reduce the possibility of misclassification, we excluded all categories except those classified as ‘diabetes unlikely’ (category 1 and 2). From those, we further excluded 1,967 participants with self-reported diabetes by TS (*diabets1*) but not confirmed by a nurse interview (*diabetes*) and diabetes medication (category 2), and additional 62 cases of gestational diabetes (derived from Data-field 20002). The final ‘diabetes unlikely’ sample consisted of 470,409 participants (Figure S1).

Counts and frequencies of *all diabetes drugs* (including metformin) in 500,234 participants are presented in Table S2.

In 470,409 ‘diabetes unlikely’ sample, 470,409 had data for grip strength, 437,850 for HbA1c, and 403,015 for random glucose. Extreme values for each measure were examined based on data distribution and boxplot inspection for extreme outliers (IBM® SPSS®, Inc.) and defined as any observations (z-score) with a threshold set at 10 for HbA1c and glucose and 5 for grip strength. Three hundred and seventy-seven participants had extreme values for glucose, 348 for HbA1c and 12 for grip strength. Of those, 273 participants had one extreme outlier, and 232 had two outliers. For grip strength, 0 kg values were kept as they indicate a true 0. In all, 505 participants were excluded as extreme outliers. At baseline, 381,715 participants had ‘diabetes unlikely’ (i.e., no prevalent diabetes) and complete data for glycaemic measure and grip strength without extreme outliers (i.e., analytic sample). Of those, 36,228 had grip strength measured at the imaging visit over a mean of 8.9 years (Figure S1).

**Grip strength and probable sarcopenia at baseline**

Grip strength (GS) was measured in each hand using a Jamar J00105 hydraulic handheld dynamometer (in kg) for a single 3-second grip force in each hand. Participants were sitting upright with the arm to their side and elbow flexed at 90° and their forearm resting on an armrest. The maximum value achieved (in kg) was used in subsequent analyses as a continuous variable and cut-offs were applied to identify probable sarcopenia (i.e., low GS; <27 kg in males and <16 kg in females as defined by the European Working Group on Sarcopenia in Older People 2 (EWGSOP2) algorithm [3].

**Grip strength patterns of change over 9 years**

Because GS was only assessed at two time points (at baseline and follow-up on average 8.9 years later; follow-up time range 3.8 to 12.8 years), we used patterns of GS change to minimise the effects of regression to the mean and measurement error as previously described in [27,28]. First, we derived age (per decade) and sex-specific Z-scores of GS at baseline and imaging visits (i.e., the number of standard deviations an outcome is above the mean for the relevant age group and sex at two points of measurement). Second, each Z-score was then grouped into three categories: low (<−1), intermediate (between −1 and 1), and high (>1). Third, change in GS between the two visits was then classified into four sex- and age-specific (per decade) patterns based on the Z-score groups: (a) low/intermediate at baseline and intermediate/high at the imaging visit (reference; referred to as ‘increase or maintained within the normal range’); (b) high at baseline and low/intermediate at imaging visit, or intermediate at baseline and low at follow-up (decline); (c) high at both visits (stable high), and (d) low at both visits (stable low) [27,28].

**Potential covariates**

**Sociodemographic factors**

Age (in years; continuous) was calculated from month and date of birth at baseline and also categorised into <50, 50–59, 60–64, and ≥65 years. Sex was self-reported as male or female. For socio-economic status, the Townsend Deprivation Index was used and categorised in fifths (Q1–Q5; Q5 (most deprived) as a reference). Years of educations were derived from the highest self-reported qualifications based on the following categories and the International Standard Classification of Education [S3: no relevant qualification (7 years), CSEs/GCSE/O-levels or equivalent (10 years), A levels/AS levels or equivalent (13 years), other professions (15 years), NVQ/HND/HNC or equivalent (19 years), and college or university degree (20 years).

Townsend deprivation index scores were derived from national census data that included car ownership, household overcrowding, owner occupation, and unemployment which were aggregated and linked to the postcodes of residence [S4]. Higher Townsend scores represent higher levels of socioeconomic deprivation.

**Health-related factors**

*Long-term conditions (LTC)*. At baseline, participants were asked if they had ever been told by a doctor that they had one or more of the following diseases: heart attack, angina, stroke, high blood pressure, blood clot in leg, blood clot in lung, emphysema/chronic bronchitis, asthma, or diabetes. Those reporting any of these conditions, or with a history of cancer, other serious LTC or disability, were then further interviewed by a research nurse who recorded details of all LTCs based on a hierarchical tree of over 450 conditions from the ICD-10 classification. The presence or absence (yes (1), no (0)) of one or more conditions in each top-level category by body systems (e.g., cardiovascular, gastrointestinal) was summarised, along with a separate variable for participants with a history of cancer(s) [28]. The total number of body systems affected by at least one LTC (excluding endocrine system/diabetes) was categorised into 0, 1, 2, and ≥3 [reference] as previously described [28] and used in the analyses.

**Lifestyle factors**

*Smoking and alcohol intake status.* Self-reported smoking and alcohol intake status was categorised as never (reference for smoking), previous, and current (reference for alcohol intake).

*Leisure time physical activity (LTPA).* LTPA comprised of five activities: walking for pleasure, light DIY (do it yourself), heavy DIY, strenuous sports, and other exercises. Participants were asked to quantify frequency and duration of participation in these activities in the last four weeks. For each, a metabolic equivalent of task (MET) value was assigned as follows: walking for pleasure (3.5), light DIY (1.5), heavy DIY (5.5), strenuous sports (8.0), and other exercises (4.0) [S5]. The total LTPA (MET-min/week) was calculated by multiplying average weekly frequency, duration and MET values and participants further categorised into three LTPA groups: low (<600 MET-min/week), moderate (600 to <3000 MET-min/week) and high (≥3000 MET-min/week; reference) as described in low (<600 MET-min/week), moderate (600 to <3000 MET-min/week) as described in [29].

*Fruit and vegetables intake*. At baseline, participants completed a dietary touchscreen questionnaire at an AC that including twenty-nine questions about diet and eight questions about alcohol. The questionnaire asked about the frequency of consumption per day over the past year of several food groups such as cooked vegetables, salad/raw vegetables, fresh fruit, and dried fruit. Participants were asked: "About how many of … would you eat per day?” followed by separate questions for pieces of fresh and dried fruit, heaped tablespoons of salad or cooked/raw vegetables [S6]. These frequencies were combined and converted to g/day (1 portion = 80 g), and further categorised into ‘healthy’ fruit and vegetables intake (≥400 g/day or ≥5 portions a day) and ‘unhealthy’ intake (<400 g/day or <5 portions a day) as in [30].

**Anthropometrics**

Height (in cm) was measured using a Seca 202 device in all participants. Body mass index (BMI) (continuous) was calculated as weight/height^2^ (kg/m^2^) and also categorised as normal (<25 kg/m^2^), overweight (25–30 kg/m^2^) and obese (>30 kg/m^2^).

**Missing data for covariates**

Missing data for covariates is presented in Table S3 for meles and females in the analytic sample (‘diabetes unlikely’ at baseline): 350,217 (91.7%) participants had no missing vales, and 31,499 (8.3%) had at least one covariate with a missing value (12,751 males and 18,747 females). Of those missing covariates, 7.9% had one (n = 30,144), 0.3% had two (n = 1,274), and the rest had 3-5 (n = 80) missing values (only one participant had five missing covariates). In sensitivity analyses, height (in cm; continuous) was missing in 474 (0.1%) participants.

**Statistical Analyses**

**Regression analyses and linearity assumptions**

Linear regression assumptions were tested separately by sex for each glycaemic measure, after examining sex x glycaemic measure (centred) and age group x glycaemic measure interaction terms in all participants in the analytic sample. Significant interaction terms provided the rationale for sex- and age-stratified analyses (Table S4 to Table S6).

To test the linearity of the associations between glycaemic measures and GS, residuals were inspected for non-linearity by examining scatter plots. Linear regression ANOVA was significant for both, and when quadratic and cubic relationship were investigated (e.g., adding HbA_1c_^2^ and HbA_1c_^3^ to the models), no improvements in R^2^ model fit were observed, thus the models were kept as linear (Table S4). The normality of residuals was tested by inspected normal probability plots (P-P). The independence of observations was tested by Durbin-Watson statistics. Homoscedasticity was determined by inspecting scatter plots for any obvious pattens in residuals, and multicollinearity in the fully adjusted models was inspected by examining VIF (variance inflation factor) <10. Descriptive statistics for grip strength and glycaemic measures by sex and across the age groups are presented in Table S5. Tests for interaction between sex, age groups and glycaemic measures in linear regression models are presented in Table S6.

**Supplementary analyses**

All multivariable regression analyses (linear, logistic, multinomial regression models) conducted for HbA1c to examine cross-sectional and longitudinal associations with GS were repeated for random glucose (Model 0: unadjusted; Model 1: adjusted for sociodemographic, health, and lifestyle factors) and reported in Appendix 2 (Table S7 to Table S9).

**Sensitivity analyses**

Several sensitivity multivariable regression analyses were conducted and compared with the main findings (Table S10 to Table S21). Fully adjusted linear, logistic, and multinomial regression models (M1) were repeated with height [S7] instead of BMI as a covariate (Table S10 to Table S12) and by excluding LTC from the models (Table S13 to Table S15) to test the robustness of the effects reported in the main results. Height is strongly associated with GS [S7] and excluding LTC from the models tested whether the associations between glycaemic measures and GS were independent of conditions linked to insulin resistance (e.g., cardiovascular diseases) [S8] as a potential mechanism of glucose effects on skeletal muscle strength and mass [39,40]. Next, key multivariable models were repeated in the analytic sample after excluding 2,417 (0.7%) participants who had HbA1c ≥48 mmol/mol (≥ 6.5%) and / or glucose ≥11.1 mmol/l (sample size n = 379,298, 54.8% female) to minimise the possibility of undiagnosed diabetes cases affecting the associations between glycaemic measures and GS (Table S16 to Table S18) [S9].

Lastly, participants missing a value in any of the eight confounders (n = 31,498, 8.3%) were excluded from the analytic sample and key regression models repeated (i.e., linear and logistic regression models for cross-sectional associations, and multinomial regression models for longitudinal associations between glycaemic measures and grip strength in males and females). The results are presented in Table S19 to Table S21.

**References**

S1. UK Biobank. UK Biobank: UK Biobank: Protocol for a large-scale prospective epidemiological resource Protocol No: UKBB-PROT-09-06 (Main Phase) 2007; 06(March): 1–112. Available from: <https://www.ukbiobank.ac.uk/media/gnkeyh2q/study-rationale.pdf>.

S2. Sudlow C, Gallacher J, Allen N, et al. UK biobank: an open access resource for identifying the causes of a wide range of complex diseases of middle and old age. PLoS Med 2015;12: e1001779.

S3. UNESCO Institute for Statistics. International Standard Classification of Education (ISCED) 2011. Available from: <https://uis.unesco.org/sites/default/files/documents/international-standard-classification-of-education-isced-2011-en.pdf>

S4. Townsend P, Philimore P, Beattie A. Health and deprivation: inequality and the North. Andover: Croom Helm, 1987.

S5. Ainsworth BE, Haskell WL, Whitt MC, et al. Compendium of physical activities: an update of activity codes and MET intensities. Med Sci Sports Exerc. 2000;32:S498–S516. doi:10.1097/00005768-200009001-00009

S6. Bradbury KE, Young HJ, Guo W, et al. Dietary assessment in UK Biobank: an evaluation of the performance of the touchscreen dietary questionnaire. J Nutr Sci. 2018;7:e6. doi:10.1017/jns.2017.66

S7. UK Biobank. Resource 1421: body composition measurement protocol. Available at: <http://biobank.ctsu.ox.ac.uk/crystal/docs/body_composition.pdf>.

S8. Kuh D, Bassey EJ, Butterworth S, et al. Grip strength, postural control, and functional leg power in a representative cohort of British men and women: associations with physical activity, health status, and socioeconomic conditions. J Gerontol A Biol Sci Med Sci. 2005;60: 224–231. doi:10.1093/gerona/60.2.224

S9. Kolb H, Kempf K, Martin S. Insulin and aging - a disappointing relationship. Front Endocrinol (Lausanne). 2023; 14:1261298. doi:10.3389/fendo.2023.1261298

**Appendix 2**

**Tables**

**Table S1.** Diabetes-related variables and codes used to identify participants with prevalent diabetes in UK Biobank (n = 500,234)

|  | Data-field 2443 (TS) | Data-field 20002 (AC) | Diabetes medication | Final code | n |
| --- | --- | --- | --- | --- | --- |
|  | *diabetes1* | *diabetes* | *All diabetes drugs* |  |  |
| 1. | 0 | 0 | 0 | 0 = diabetes unlikely | 470,471 |
| 2. | 1 | 0 | 0 | 0 = diabetes unlikely | 1,967 |
| 3. | 0 | 1 | 0 | 1 = diabetes likely | 1,020 |
| 4. | 0 | 0 | 1 | 1 = diabetes likely | 316 |
| 5. | 1 | 1 | 0 | 1 = diabetes likely | 5,714 |
| 6. | 1 | 0 | 1 | 1 = diabetes likely | 2,612 |
| 7. | 0 | 1 | 1 | 1 = diabetes likely | 14 |
| 8. | 1 | 1 | 1 | 1 = diabetes likely | 15,951 |
|  |  |  |  | Missing | 2,169 |
|  |  |  |  | Total | 500,234 |

TS, touch screen; AC, Assessment Centre

**Table S2.** All diabetes drugs intake in 500,234 UK Biobank participants at baseline

| **Count** | **Frequency (n)** | **%** | **Description** |
| --- | --- | --- | --- |
| 0 | 481,211 | 97.6 | Take 0 drugs or data missing |
| 1 | 10,135 | 2.0 | Take 1 diabetes drug |
| 2 | 1,762 | 0.4 | Take 2 diabetes drugs |
| 3 | 122 | 0 | Take 3 diabetes drugs |
| 4 | 4 | 0 | Take 4 diabetes drugs |
|  |  |  |  |
|  |  |  | Total: 500,234 |
| *metformin* | | | |
| code 0 | 486,379 | 97.2 | no, takes other diabetes medication, or data missing |
| code 1 | 13,855 | 2.8 | yes |

**Table S3.** Missing values for eight covariates in the analytic sample at baseline in UK Biobank

| **Covariate** | **Males** | **Females** |
| --- | --- | --- |
| n (%) | 172,795 (45.3) | 208,920 (54.7) |
| Age, years, n (%) | 0 (0) | 0 (0) |
| Education, years, n (%) | 1,774 (1) | 2,034 (1) |
| Townsend deprivation index, n (%) | 311 (0.2) | 355 (0.2) |
| Long-term conditions (no diabetes), n (%) | 2 (0) | 6 (0) |
| BMI, kg/m^2^, n (%) | 462 (0.3) | 394 (0.2) |
| Smoking status, n (%) | 626 (0.4) | 743 (0.4) |
| Alcohol intake status, n (%) | 185 (0.1) | 217 (0.1) |
| Leisure-time PA (MET-min/week), n (%) | 10,406 (6) | 16,364 (7.8) |
| Fruit and vegetable intake, g/day, n (%) | 314 (0.2) | 139 (0.1) |

**Table S4.** Linearity test and curve estimations (linear, quadratic, cubed) for the association between HbA1c and grip strength in the analytic sample in UK Biobank

| **Block/model** | **Adjusted R^2^** | **F change** | **R^2^ change** |
| --- | --- | --- | --- |
| Model 1 | 0.005 | 1872.707 | 0.005 |
| Model 2 | 0.005 | 235.961 | 0.001 |
| Model 3 | 0.006 | 83.307 | 0.000 |

Model 1 includes HbA1c (linear). Model 2 is additionally adjusted for HbA1c^2^. Model 3 is further adjusted for HbA1c^3^.

**Table S5.** Grip strength and glycaemic measures in participants across the age groups in the analytic sample at baseline in UK Biobank (n = 381,715)

|  |  | **Males** | | |  | **Females** | | |
| --- | --- | --- | --- | --- | --- | --- | --- | --- |
| Age group (years) | *N* | GS (SD), kg | HbA1c (SD), mmol/mol | Glucose (SD), mmol/l | *N* | GS (SD), kg | HbA1c (SD), mmol/mol | Glucose (SD), mmol/l |
| <50 | 41,747 | 45.2 (9.3) | 34.0 (4.0) | 4.9 (0.7) | 49,776 | 28.2 (6.3) | 33.1 (3.6) | 4.8 (0.6) |
| 50-59 | 55,559 | 42.9 (8.7) | 35.0 (4.1) | 5.0 (0.8) | 72,035 | 25.5 (7.1) | 35.1 (3.8) | 4.9 (0.7) |
| 60-64 | 41,219 | 40.5 (8.3) | 35.7 (4.1) | 5.0 (0.7) | 50,589 | 23.7 (5.9) | 36.0 (3.7) | 5.0 (0.7) |
| ≥65 | 34,270 | 38.4 (8.0) | 36.2 (4.2) | 5.1 (0.7) | 36,520 | 22.4 (5.8) | 36.6 (3.7) | 5.1 (0.7) |

SD, standard deviation.

**Table S6.** Test for interactions between sex, age groups, and glycaemic measures in regression models in the analytic sample in UK Biobank (n = 381,715)

| **Block/Model** | **Regression coefficient** | **95% CI** | ***p-*value** |
| --- | --- | --- | --- |
| HbA1c^a^ | -0.21 | -0.22, -0.21 | <0.001 |
| sex | 16.86 | 16.81, 16.91 | <0.001 |
| sex x HbA1c | 0.02 | 0.005, 0.03 | 0.004 |
|  |  |  |  |
| Glucose^a^ | -0.55 | -0.60, -0.50 | <0.001 |
| sex | 16.86 | 16.81, 16.90 | <0.001 |
| sex x glucose | 0.03 | -0.04, 0.10 | 0.4 |
|  |  |  |  |
| HbA1c | 0.07 | 0.05, 0.9 | <0.001 |
| age groups | -1.88 | -1.92, -1.85 | <0.001 |
| HbA1c x age groups | -0.06 | -0.7, -0.05 | <0.001 |
|  |  |  |  |
| Glucose | 0.53 | 0.40, 0.67 | <0.001 |
| age groups | 1.95 | -1.99, -1.92 | <0.001 |
| age groups x glucose | -0.23 | -0.28, -0.18 | <0.001 |

^a^HbA1c and glucose were mean centred.

**Table S7.** Associations between random glucose and grip strength in participants without prevalent diabetes at baseline in UK Biobank (n = 381,715)

| **Males** | | | | **Females** | | | |
| --- | --- | --- | --- | --- | --- | --- | --- |
| **Glucose (mmol/l)** | | | | | | | |
| **Model** | **Regression coefficient^a^** | **95% CI** | ***p-*value** | **Model** | **Regression coefficient^a^** | **95% CI** | ***p-*value** |
| M0 | -0.52 | -0.58, -0.46 | <0.001 | M0 | -0.55 | -0.59, -0.51 | <0.001 |
| M1 | -0.19 | -0.25, -0.14 | <0.001 | M1 | 0.03 | -0.01, 0.07 | 0.17 |
| **Age group** |  |  |  | **Age group** |  |  |  |
| **<50 years** |  |  |  | **<50 years** |  |  |  |
| M0 | -0.07 | -0.25, 0.02 | 0.08 | M0 | -0.03 | -0.12, 0.06 | 0.55 |
| M1 | -0.27 | -0.40, -0.14 | <0.001 | M1 | -0.02 | -0.09, 0.09 | 0.97 |
| **50-59 years** |  |  |  | **50-59 years** |  |  |  |
| M0 | -0.21 | -0.32, -0.11 | <0.001 | M0 | -0.19 | -0.26, -0.12 | <0.001 |
| M1 | -0.26 | -0.36, -0.15 | <0.001 | M1 | -0.12 | -0.2, -0.05 | <0.001 |
| **60-64 years** |  |  |  | **60-64 years** |  |  |  |
| M0 | -0.19 | -0.31, -0.08 | <0.001 | M0 | 0.02 | -0.06, 0.10 | 0.62 |
| M1 | -0.21 | -0.32, -0.1 | <0.001 | M1 | 0.06 | -0.02, 0.13 | 0.13 |
| **≥65 years** |  |  |  | **≥65 years** |  |  |  |
| M0 | -0.12 | -0.24, -0.01 | 0.03 | M0 | 0.09 | 0.01, 0.18 | 0.03 |
| M1 | -0.11 | -0.23, -0.001 | 0.06 | M1 | 0.11 | 0.03, 0.20 | 0.01 |

GS, grip strength. ^a^Regression coefficients represent the difference in mean GS (in kg) per 1 unit change in glycaemic measure.

Model 0 (M0) is unadjusted.

Model 1 (M1) is adjusted for age (in all), years of education, Townsend index of deprivation, number of long-term conditions, and BMI, leisure-time physical activity, smoking status, alcohol drinking status, and fruit and vegetable intake.

**Table S8.** Odds ratios and 95% CIs of the associations between random glucose and grip strength in participants without prevalent diabetes at baseline in UK Biobank (n = 381,715)

| **Males** | | | **Females** | | |
| --- | --- | --- | --- | --- | --- |
| **Glucose (mmol/l)** | | | | | |
| **Model** | **OR (95% CI)** | ***p-*value** | **Model** | **OR (95% CI)** | ***p-*value** |
| M0 | 1.13 (1.09, 1.16) | <0.001 | M0 | 1.18 (1.15, 1.21) | <0.001 |
| M1 | 1.05 (1.02, 1.09) | 0.002 | M1 | 1.02 (1.00, 1.04) | 0.29 |
| **Age group** |  |  | **Age group** |  |  |
| **<50 years** |  |  | **<50 years** |  |  |
| M0 | 1.16 (1.07, 1.26) | <0.001 | M0 | 1.03 (0.94, 1.14) | 0.5 |
| M1 | 1.14 (1.05, 1.24) | 0.002 | M1 | 0.97 (0.88, 1.07) | 0.53 |
| **50-59 years** |  |  | **50-59 years** |  |  |
| M0 | 1.15 (1.08, 1.22) | <0.001 | M0 | 1.15 (1.10, 1.21) | <0.001 |
| M1 | 1.13 (1.06, 1.20) | <0.001 | M1 | 1.09 (1.03, 1.14) | 0.001 |
| **60-64 years** |  |  | **60-64 years** |  |  |
| M0 | 1.06 (1.00, 1.13) | 0.07 | M0 | 1.04 (1.00, 1.1) | 0.08 |
| M1 | 1.04 (0.98, 1.11) | 0.21 | M1 | 1.01 (0.96, 1.06) | 0.62 |
| **≥65 years** |  |  | **≥65 years** |  |  |
| M0 | 1.00 (0.94, 1.06) | 0.9 | M0 | 0.99 (0.95, 1.04) | 0.73 |
| M1 | 1.00 (0.92, 1.04) | 0.48 | M1 | 0.98 (0.93, 1.03) | 0.36 |

Model 0 is unadjusted.

Model 1 adjusted for age (in all), years of education, Townsend index of deprivation, number of long-term conditions, BMI, leisure-time physical activity, smoking status, alcohol intake status, and fruit and vegetable intake.

**Table S9.** Associations between random glucose and patterns of grip strength change in participants without prevalent diabetes at baseline in UK Biobank (n = 36,228)

| **All** | | | **Male** | | **Female** | |
| --- | --- | --- | --- | --- | --- | --- |
| **n = 36,228** | | | **n = 17,451** | | **n = 18,777** | |
| **GS pattern^a^** | **OR (95% CI)^b^** | ***p-*value** | **OR (95% CI)^b^** | ***p-*value** | **OR (95% CI)^b^** | ***p-*value** |
| **Decline** | 0.94 (0.90, 0.99) | 0.01 | 0.96 (0.90, 1.02) | 0.2 | 0.93 (0.87, 0.99) | 0.02 |
| **Stable high** | 0.99 (0.93, 1.06) | 0.75 | 0.99 (0.91, 1.09) | 0.88 | 0.98 (0.88, 1.08) | 0.66 |
| **Stable low** | 0.93 (0.90, 1.03) | 0.28 | 1.01 (0.92, 1.11) | 0.87 | 0.91 (0.82, 1.01) | 0.09 |

^a^GS (grip strength) pattern ‘increase or maintained within the normal range’ (reference). ^b^Multinominal regression models; OR (odds ratio) of belonging to a GS pattern per unit increase in random glucose (mmol/l).

Models are adjusted for years of education, Townsend index of deprivation, number of long-term conditions, and BMI, leisure-time physical activity, smoking status, alcohol drinking status, and fruit and vegetable intake.

**Table S10.** Associations between glycaemic measures and grip strength with height as a covariate in the analytic sample in UK Biobank (n = 381,715)

| **Males** | | | | **Females** | | | |
| --- | --- | --- | --- | --- | --- | --- | --- |
| **HbA1c (mmol/mol)** | | | | | | | |
| **All (n = 172,795)** | | | | **All (n = 208,920)** | | | |
| **Model** | **Regression coefficient^a^** | **95% CI** | ***p-*value** | **Model** | **Regression coefficient^a^** | **95% CI** | ***p-*value** |
| M1 | -0.03 | -0.04, -0.02 | <0.001 | M1 | -0.003 | -0.01, -0.004 | <0.001 |
| **Age group** |  |  |  | **Age group** |  |  |  |
| **<50 years** |  |  |  | **<50 years** |  |  |  |
| M1 | -0.04 | -0.06, -0.02 | <0.001 | M1 | -0.04 | -0.05, -0.02 | <0.001 |
| **50-59 years** |  |  |  | **50-59 years** |  |  |  |
| M1 | -0.02 | -0.04, -0.01 | 0.005 | M1 | -0.03 | -0.04, -0.02 | <0.001 |
| **60-64 years** |  |  |  | **60-64 years** |  |  |  |
| M1 | -0.04 | -0.06, -0.02 | <0.001 | M1 | 0.02 | 0.01, 0.04 | 0.001 |
| **≥65 years** |  |  |  | **≥65 years** |  |  |  |
| M1 | -0.03 | -0.05, -0.01 | <0.001 | M1 | 0.02 | 0.001, 0.03 | 0.04 |
| **Glucose (mmol/l)** | | | | | | | |
| **Model** | **Regression coefficient^a^** | **95% CI** | ***p-*value** | **Model** | **Regression coefficient^a^** | **95% CI** | ***p-*value** |
| M1 | -0.04 | -0.09, -0.02 | 0.18 | M1 | 0.05 | 0.01, -0.09 | 0.007 |
| **Age group** |  |  |  | **Age group** |  |  |  |
| **<50 years** |  |  |  | **<50 years** |  |  |  |
| M1 | -0.06 | -0.19, -0.07 | 0.37 | M1 | 0.05 | -0.04, 0.13 | 0.29 |
| **50-59 years** |  |  |  | **50-59 years** |  |  |  |
| M1 | -0.07 | -0.17, -0.03 | 0.16 | M1 | -0.08 | -0.14, -0.01 | 0.02 |
| **60-64 years** |  |  |  | **60-64 years** |  |  |  |
| M1 | -0.07 | -0.18, -0.03 | 0.18 | M1 | 0.07 | -0.01, 0.14 | 0.07 |
| **≥65 years** |  |  |  | **≥65 years** |  |  |  |
| M1 | -0.02 | -0.13, -0.09 | 0.78 | M1 | 0.14 | 0.06, 0.22 | <0.001 |

^a^Linear regression models. Model 1 adjusted for age (in all), years of education, Townsend index of deprivation, number of long-term conditions, height, leisure-time physical activity, smoking status, alcohol intake status, and fruit and vegetable intake.

**Table S11.** Odds ratios and 95% CIs of the associations between glycaemic measures and grip strength with height as covariate in participants without prevalent diabetes at baseline in UK Biobank (n = 381,715)

| **Males** | | | **Females** | | |
| --- | --- | --- | --- | --- | --- |
| **HbA1c (mmol/mol)** | | | | | |
| **Model** | **OR (95% CI)** | ***p-*value** | **Model** | **OR (95% CI)** | ***p-*value** |
| M1 | 1.01 (1.00, 1.01) | 0.004 | M1 | 1.00 (1.00, 1.01) | 0.24 |
| **Age group** |  |  | **Age group** |  |  |
| **<50 years** |  |  | **<50 years** |  |  |
| M1 | 1.01 (1.00, 1.03) | 0.07 | M1 | 1.02 (1.01, 1.04) | 0.01 |
| **50-59 years** |  |  | **50-59 years** |  |  |
| M1 | 1.01 (1.00, 1.02) | 0.03 | M1 | 1.01 (1.00, 1.02) | 0.09 |
| **60-64 years** |  |  | **60-64 years** |  |  |
| M1 | 1.01 (0.99, 1.02) | 0.34 | M1 | 0.99 (0.98, 1.00) | 0.09 |
| **≥65 years** |  |  | **≥65 years** |  |  |
| M1 | 1.01 (1.00, 1.02) | 0.19 | M1 | 1.00 (1.00, 1.01) | 0.44 |
| **Glucose (mmol/l)** | | | | | |
| **Model** | **OR (95% CI)** | ***p-*value** | **Model** | **OR (95% CI)** | ***p-*value** |
| M1 | 1.04 (1.01, 1.08) | 0.02 | M1 | 1.02 (1.00, 1.04) | 0.27 |
| **Age group** |  |  | **Age group** |  |  |
| **<50 years** |  |  | **<50 years** |  |  |
| M1 | 1.13 (1.03, 1.23) | 0.006 | M1 | 0.98 (0.89, 1.08) | 0.68 |
| **50-59 years** |  |  | **50-59 years** |  |  |
| M1 | 1.11 (1.04, 1.18) | 0.001 | M1 | 1.08 (1.03, 1.13) | 0.002 |
| **60-64 years** |  |  | **60-64 years** |  |  |
| M1 | 1.03 (0.97, 1.10) | 0.37 | M1 | 1.02 (0.97, 1.07) | 0.51 |
| **≥65 years** |  |  | **≥65 years** |  |  |
| M1 | 0.97 (0.91, 1.03) | 0.27 | M1 | 0.97 (0.93, 1.02) | 0.25 |

Model 1 adjusted for age (in all), years of education, Townsend index of deprivation, number of long-term conditions, height, leisure-time physical activity, smoking status, alcohol intake status, and fruit and vegetable intake.

**Table S12.** Associations between glycaemic measures and patterns of grip strength change with height as a covariate in participants without prevalent diabetes at baseline in UK Biobank (n = 36,228)

| **All** | | | | **Males** | | | | **Females** | | | |
| --- | --- | --- | --- | --- | --- | --- | --- | --- | --- | --- | --- |
| **n = 36,228** | | | | **n = 17,451** | | | | **n = 18,777** | | | |
| **GS pattern^a^** | **HbA1c**  **(mmol/mol)** | **OR**  **(95% CI)^b^** | ***p-*value** | **GS patten^a^** | **HbA1c**  **(mmol/mol)** | **OR**  **(95% CI)^b^** | ***p-*value** | **GS pattern^a^** | **HbA1c**  **(mmol/mol)** | **OR**  **(95% CI)^b^** | ***p-*value** |
| **Decline** |  | 0.99 (0.98, 1.00) | 0.03 | **Decline** |  | 1.00 (0.99, 1.01) | 0.75 | **Decline** |  | 0.98 (0.97, 0.99) | 0.001 |
| **Stable high** |  | 0.99 (0.96, 1.00) | 0.03 | **Stable high** |  | 0.98 (0.97, 1.00) | 0.02 | **Stable high** |  | 1.00 (0.99, 1.02) | 0.77 |
| **Stable low** |  | 1.01 (1.00, 1.03) | 0.02 | **Stable low** |  | 1.02 (1.00, 1.04) | 0.01 | **Stable low** |  | 1.00 (0.98, 1.02) | 0.87 |
| **GS pattern^a^** | **Glucose**  **(mmol/l)** | **OR**  **(95% CI)^b^** | ***p-* value** | **GS pattern^a^** | **Glucose**  **(mmol/l)** | **OR**  **(95% CI)^b^** | ***p-*value** | **GS pattern^a^** | **Glucose**  **(mmol/l)** | **OR**  **(95% CI)^b^** | ***p-*value** |
| **Decline** |  | 0.95 (0.91, 1.00) | 0.04 | **Decline** |  | 0.98 (0.91, 1.04) | 0.44 | **Decline** |  | 0.93 (0.87, 0.99) | 0.03 |
| **Stable high** |  | 1.01 (0.95, 1.08) | 0.70 | **Stable high** |  | 1.03 (0.95, 1.13) | 0.47 | **Stable high** |  | 1.01 (0.92, 1.12) | 0.78 |
| **Stable low** |  | 0.96 (0.90, 1.03) | 0.25 | **Stable low** |  | 0.99 (0.90, 1.09) | 0.77 | **Stable low** |  | 0.90 (0.81, 1.00) | 0.04 |

^a^GS (grip strength) pattern ‘increase/maintained within the normal range’ (reference). **^b^**Multinominal regression models.

Model 1 adjusted for years of education, Townsend index of deprivation, number of long-term conditions, and height, leisure-time physical activity, smoking status, alcohol drinking status, and fruit and vegetable intake.

**Table S13.** Associations between glycaemic measures and grip strength without long-term conditions as a covariate in the analytic sample in UK Biobank (n = 381,715)

| **Males** | | | | **Females** | | | |
| --- | --- | --- | --- | --- | --- | --- | --- |
| **HbA1c (mmol/mol)** | | | | | | | |
| **All (*N* = 172,795)** | | | | **All (*N* = 208,920)** | | | |
| **Model** | **Regression coefficient^a^** | **95% CI** | ***p-*value** | **Model** | **Regression coefficient^a^** | **95% CI** | ***p-*value** |
| M1 | -0.09 | -0.1, -0.08 | <0.001 | M1 | -0.03 | -0.03, -0.02 | <0.001 |
| **Age group** |  |  |  | **Age group** |  |  |  |
| **<50 years** |  |  |  | **<50 years** |  |  |  |
| M1 | -0.12 | -0.15, -0.10 | <0.001 | M1 | -0.07 | -0.08, -0.05 | <0.001 |
| **50-59 years** |  |  |  | **50-59 years** |  |  |  |
| M1 | -0.09 | -0.11, -0.08 | <0.001 | M1 | -0.07 | -0.08, -0.05 | <0.001 |
| **60-64 years** |  |  |  | **60-64 years** |  |  |  |
| M1 | -0.09 | -0.11, -0.07 | <0.001 | M1 | 0.002 | 0.01, 0.02 | 0.77 |
| **≥65 years** |  |  |  | **≥65 years** |  |  |  |
| M1 | -0.07 | -0.09, -0.05 | <0.001 | M1 | -0.01 | -0.02, 0.01 | 0.49 |
| **Glucose (mmol/l)** | | | | | | | |
| **Model** | **Regression coefficient^a^** | **95% CI** | ***p-*value** | **Model** | **Regression coefficient^a^** | **95% CI** | ***p-*value** |
| M1 | -0.21 | -0.27, -0.15 | <0.001 | M1 | 0.01 | -0.03, 0.05 | 0.56 |
| **Age group** |  |  |  | **Age group** |  |  |  |
| **<50 years** |  |  |  | **<50 years** |  |  |  |
| M1 | -0.28 | -0.41, -0.15 | <0.001 | M1 | -0.01 | -0.10, 0.08 | 0.85 |
| **50-59 years** |  |  |  | **50-59 years** |  |  |  |
| M1 | -0.28 | -0.38, -0.18 | <0.001 | M1 | -0.15 | -0.22, -0.08 | <0.001 |
| **60-64 years** |  |  |  | **60-64 years** |  |  |  |
| M1 | -0.23 | -0.34, -0.11 | <0.001 | M1 | 0.04 | -0.04, 0.12 | 0.29 |
| **≥65 years** |  |  |  | **≥65 years** |  |  |  |
| M1 | -0.13 | -0.25, -0.02 | 0.03 | M1 | 0.09 | 0.01, 0.18 | 0.03 |

^a^Linear regression models. Model 1 adjusted for age (in all), years of education, Townsend index of deprivation, BMI, leisure-time physical activity, smoking status, alcohol intake status, and fruit and vegetable intake.

**Table S14.** Odds ratios and 95% CIs of the associations between glycaemic measures and grip strength without long-term conditions as covariate in participants without prevalent diabetes at baseline in UK Biobank (n = 381,715)

| **Males** | | | **Females** | | |
| --- | --- | --- | --- | --- | --- |
| **HbA1c (mmol/mol)** | | | | | |
| **Model** | **OR (95% CI)** | ***p-*value** | **Model** | **OR (95% CI)** | ***p-*value** |
| M1 | 1.02 (1.00, 1.01) | <0.001 | M1 | 1.01 (1.00, 1.01) | 0.005 |
| **Age group** |  |  | **Age group** |  |  |
| **<50 years** |  |  | **<50 years** |  |  |
| M1 | 1.03 (1.01, 1.04) | <0.001 | M1 | 1.02 (1.00, 1.04) | 0.01 |
| **50-59 years** |  |  | **50-59 years** |  |  |
| M1 | 1.02 (1.01, 1.04) | <0.001 | M1 | 1.02 (1.01, 1.02) | <0.001 |
| **60-64 years** |  |  | **60-64 years** |  |  |
| M1 | 1.01 (1.00, 1.03) | 0.01 | M1 | 1.00 (0.99, 1.01) | 0.34 |
| **≥65 years** |  |  | **≥65 years** |  |  |
| M1 | 1.01 (1.00, 1.02) | 0.01 | M1 | 1.01 (1.00, 1.02) | 0.04 |
| **Glucose (mmol/l)** | | | | | |
| **Model** | **OR (95% CI)** | ***p*-value** | **Model** | **OR (95% CI)** | ***p*-value** |
| M1 | 1.06 (1.02, 1.09) | <0.001 | M1 | 1.03 (1.00, 1.05) | 0.07 |
| **Age group** |  |  | **Age group** |  |  |
| **<50 years** |  |  | **<50 years** |  |  |
| M1 | 1.15 (1.06, 1.25) | 0.002 | M1 | 0.98 (0.88, 1.08) | 0.63 |
| **50-59 years** |  |  | **50-59 years** |  |  |
| M1 | 1.14 (1.07, 1.21) | <0.001 | M1 | 1.10 (1.05, 1.16) | <0.001 |
| **60-64 years** |  |  | **60-64 years** |  |  |
| M1 | 1.05 (0.98, 1.11) | 0.18 | M1 | 1.02 (0.97, 1.07) | 0.36 |
| **≥65 years** |  |  | **≥65 years** |  |  |
| M1 | 0.98 (0.93, 1.04) | 0.57 | M1 | 0.99 (0.94, 1.04) | 0.57 |

Model 1 adjusted for age (in all), years of education, Townsend index of deprivation, BMI, leisure-time physical activity, smoking status, alcohol intake status, and fruit and vegetable intake.

**Table S15.** Multinomial regression associations between glycaemic measures and patterns of grip strength change without long-term conditions as a covariate in participants without prevalent diabetes at baseline in UK Biobank (n = 36,228)

| **All** | | | | **Males** | | | | **Females** | | | |
| --- | --- | --- | --- | --- | --- | --- | --- | --- | --- | --- | --- |
| **n = 36,228** | | | | **n = 17,451** | | | | **n = 18,777** | | | |
| **GS pattern^a^** | **HbA1c**  **(mmol/mol)** | **OR**  **(95% CI)^b^** | ***p-*value** | **GS patten^a^** | **HbA1c**  **(mmol/mol)** | **OR**  **(95% CI)^b^** | ***p-*value** | **GS pattern^a^** | **HbA1c**  **(mmol/mol)** | **OR**  **(95% CI)^b^** | ***p-*value** |
| **Decline** |  | 0.99 (0.98, 1.00) | 0.005 | **Decline** |  | 1.00 (0.99, 1.01) | 0.79 | **Decline** |  | 0.98 (0.97, 0.99) | <0.001 |
| **Stable high** |  | 0.98 (0.97, 0.99) | <0.001 | **Stable high** |  | 0.97 (0.96, 0.99) | <0.001 | **Stable high** |  | 0.99 (0.97, 1.00) | 0.12 |
| **Stable low** |  | 1.02 (1.01, 1.03) | 0.001 | **Stable low** |  | 1.02 (1.00, 1.04) | <0.001 | **Stable low** |  | 1.01 (0.99, 1.03) | 0.23 |
| **GS pattern^a^** | **Glucose**  **(mmol/l)** | **OR**  **(95% CI)^b^** | ***p-*value** | **GS pattern^a^** | **Glucose**  **(mmol/l)** | **OR**  **(95% CI)^b^** | ***p-*value** | **GS pattern^a^** | **Glucose**  **(mmol/l)** | **OR**  **(95% CI)^b^** | ***p-*value** |
| **Decline** |  | 0.94 (0.90, 0.99) | 0.01 | **Decline** |  | 0.96 (0.80, 1.02) | 0.21 | **Decline** |  | 0.93 (0.87, 0.99) | 0.03 |
| **Stable high** |  | 0.99 (0.92, 1.06) | 0.71 | **Stable high** |  | 0.99 (0.91, 1.09) | 0.87 | **Stable high** |  | 0.98 (0.88, 1.08) | 0.65 |
| **Stable low** |  | 0.97 (0.91, 1.04) | 0.97 | **Stable low** |  | 1.01 (0.92, 1.11) | 0.85 | **Stable low** |  | 0.93 (0.84, 1.03) | 0.14 |

^a^GS (grip strength) pattern ‘increase/maintained within the normal range’ (reference). ^b^Multinominal regression models.

Model 1 adjusted for years of education, Townsend index of deprivation, BMI, leisure-time physical activity, smoking status, alcohol drinking status, and fruit and vegetable intake.

**Table S16.** Associations between glycaemic measures and grip strength in participants without prevalent diabetes and excluding those with HbA1c ≥48 mmol/mol and/or glucose >11.1 mmol/l at baseline in UK Biobank (n = 379,298)

| **Males** | | | | **Females** | | | |
| --- | --- | --- | --- | --- | --- | --- | --- |
| **HbA1c (mmol/mol)** | | | | | | | |
| **All (n = 171,394)** | | | | **All (n = 207,904)** | | | |
| **Model** | **Regression coefficient^a^** | **95% CI** | ***p-*value** | **Model** | **Regression coefficient^a^** | **95% CI** | ***p-*value** |
| M1 | -0.08 | -0.09, -0.07 | <0.001 | M1 | -0.02 | -0.03, -0.01 | <0.001 |
| **Age group** |  |  |  | **Age group** |  |  |  |
| **<50 years** |  |  |  | **<50 years** |  |  |  |
| M1 | -0.11 | -0.14, -0.09 | <0.001 | M1 | -0.07 | -0.08, -0.05 | <0.001 |
| **50-59 years** |  |  |  | **50-59 years** |  |  |  |
| M1 | -0.09 | -0.11, -0.07 | <0.001 | M1 | -0.06 | -0.07, -0.04 | <0.001 |
| **60-64 years** |  |  |  | **60-64 years** |  |  |  |
| M1 | -0.08 | -0.11, -0.06 | <0.001 | M1 | 0.01 | -0.001, 0.03 | 0.07 |
| **≥65 years** |  |  |  | **≥65 years** |  |  |  |
| M1 | -0.07 | -0.09, -0.05 | <0.001 | M1 | -0.01 | -0.02, 0.01 | 0.59 |
| **Glucose (mmol/l)** | | | | | | | |
| **Model** | **Regression coefficient^a^** | **95% CI** | ***p-*value** | **Model** | **Regression coefficient^a^** | **95% CI** | ***p-*value** |
| M1 | -0.17 | -0.23, -0.11 | <0.001 | M1 | 0.05 | 0.01, -0.09 | 0.01 |
| **Age group** |  |  |  | **Age group** |  |  |  |
| **<50 years** |  |  |  | **<50 years** |  |  |  |
| M1 | -0.21 | -0.35, -0.07 | 0.004 | M1 | 0.01 | 0.09, 0.10 | 0.89 |
| **50-59 years** |  |  |  | **50-59 years** |  |  |  |
| M1 | -0.24 | -0.35, -0.13 | <0.001 | M1 | -0.09 | -0.16, -0.02 | 0.01 |
| **60-64 years** |  |  |  | **60-64 years** |  |  |  |
| M1 | -0.19 | -0.31, -0.07 | 0.002 | M1 | 0.08 | -0.003, -0.15 | 0.06 |
| **≥65 years** |  |  |  | **≥65 years** |  |  |  |
| M1 | -0.11 | -0.24, 0.01 | 0.08 | M1 | 0.14 | 0.05, 0.22 | 0.002 |

^a^Linear regression models. Model 1 (M1) is adjusted for age (in all), years of education, Townsend index of deprivation, number of long-term conditions, and BMI, leisure-time physical activity, smoking status, alcohol drinking status, and fruit and vegetable intake.

**Table S17.** Odds ratios between glycemic measures and probable sarcopenia in participants without prevalent diabetes and excluding those with HbA1c ≥48 mmol/mol and/or glucose >11.1 mmol/l at baseline in UK Biobank (n = 379,298)

| **Males** | | | **Females** | | |
| --- | --- | --- | --- | --- | --- |
| **HbA1c (mmol/mol)** | | | | | |
| **Model** | **OR (95% CI)** | ***p-*value** | **Model** | **OR (95% CI)** | ***p-*value** |
| M1 | 1.02 (1.01, 1.02) | <0.001 | M1 | 1.00 (1.00, 1.01) | 0.18 |
| **Age group** |  |  | **Age group** |  |  |
| **<50 years** |  |  | **<50 years** |  |  |
| M1 | 1.03 (1.01, 1.05) | 0.003 | M1 | 1.02 (1.00, 1.04) | 0.02 |
| **50-59 years** |  |  | **50-59 years** |  |  |
| M1 | 1.02 (1.01, 1.04) | 0.002 | M1 | 1.01 (1.00, 1.02) | 0.24 |
| **60-64 years** |  |  | **60-64 years** |  |  |
| M1 | 1.01 (1.00, 1.03) | 0.08 | M1 | 0.99 (0.98, 1.00) | 0.18 |
| **≥65 years** |  |  | **≥65 years** |  |  |
| M1 | 1.02 (1.00, 1.03) | 0.007 | M1 | 1.01 (1.00, 1.02) | 0.08 |
| **Glucose (mmol/l)** | | | | | |
| **Model** | **OR (95% CI)** | ***p-*value** | **Model** | **OR (95% CI)** | ***p-*value** |
| M1 | 1.06 (1.02, 1.10) | 0.002 | M1 | 1.00 (0.98, 1.03) | 0.77 |
| **Age group** |  |  | **Age group** |  |  |
| **<50 years** |  |  | **<50 years** |  |  |
| M1 | 1.17 (1.06, 1.29) | 0.002 | M1 | 0.96 (0.87, 1.07) | 0.47 |
| **50-59 years** |  |  | **50-59 years** |  |  |
| M1 | 1.13 (1.05, 1.21) | <0.001 | M1 | 1.06 (1.00, 1.12) | 0.04 |
| **60-64 years** |  |  | **60-64 years** |  |  |
| M1 | 1.05 (0.98, 1.13) | 0.18 | M1 | 1.03 (0.97, 1.08) | 0.34 |
| **≥65 years** |  |  | **≥65 years** |  |  |
| M1 | 0.99 (0.93, 1.06) | 0.81 | M1 | 0.96 (0.92, 1.02) | 0.16 |

Model 1 (M1) is adjusted for age (in all), years of education, Townsend index of deprivation, number of long-term conditions, and BMI, leisure-time physical activity, smoking status, alcohol drinking status, and fruit and vegetable intake.

**Table S18.** Associations between glycaemic measures and patterns of grip strength change in participants without prevalent diabetes at baseline and excluding those with HbA1c ≥48 mmol/mol and/or glucose >11.1 mmol/l at baseline in UK Biobank (n = 36,151)

| **All** | | | | **Males** | | | | **Females** | | | |
| --- | --- | --- | --- | --- | --- | --- | --- | --- | --- | --- | --- |
| **n = 36,151** | | | | **n = 18,743** | | | | **n = 17,408** | | | |
| **GS pattern^a^** | **HbA1c**  **(mmol/mol)** | **OR**  **(95% CI)^b^** | ***p-*value** | **GS patten^a^** | **HbA1c**  **(mmol/mol)** | **OR**  **(95% CI)^b^** | ***p-*value** | **GS pattern^a^** | **HbA1c**  **(mmol/mol)** | **OR**  **(95% CI)^b^** | ***p-*value** |
| **Decline** |  | 0.99 (0.98, 1.00) | 0.002 | **Decline** |  | 1.00 (0.99, 1.01) | 0.73 | **Decline** |  | 0.98 (0.97, 0.99) | <0.001 |
| **Stable high** |  | 0.97 (0.96, 0.99) | <0.001 | **Stable high** |  | 0.97 (0.95, 0.98) | <0.001 | **Stable high** |  | 0.98 (0.97, 1.00) | 0.07 |
| **Stable low** |  | 1.02 (1.00, 1.03) | 0.01 | **Stable low** |  | 1.03 (1.01, 1.04) | 0.004 | **Stable low** |  | 1.01 (0.99, 1.02) | 0.49 |
| **GS pattern^a^** | **Glucose**  **(mmol/l)** | **OR**  **(95% CI)^b^** | ***p-*value** | **GS pattern^a^** | **Glucose**  **(mmol/l)** | **OR**  **(95% CI)^b^** | ***p-*value** | **GS pattern^a^** | **Glucose**  **(mmol/l)** | **OR**  **(95% CI)^b^** | ***p-*value** |
| **Decline** |  | 0.94 (0.90, 0.99) | 0.01 | **Decline** |  | 0.96 (0.89, 1.02) | 0.18 | **Decline** |  | 0.93 (0.87, 0.99) | 0.02 |
| **Stable high** |  | 0.98 (0.91, 1.05) | 0.48 | **Stable high** |  | 0.97 (0.88, 1.07) | 0.51 | **Stable high** |  | 0.98 (0.88, 1.08) | 0.65 |
| **Stable low** |  | 0.96 (0.89, 1.03) | 0.22 | **Stable low** |  | 0.99 (0.90, 1.10) | 0.91 | **Stable low** |  | 0.92 (0.83, 1.02) | 0.10 |

^a^GS (grip strength) pattern ‘increase/maintained within the normal range’ (reference). ^b^Multinominal regression models.

Model is adjusted for age, years of education, Townsend index of deprivation, number of long-term conditions, and BMI, leisure-time physical activity, smoking status, alcohol drinking status, and fruit and vegetable intake.

**Table S19.** Associations between glycaemic measures and grip strength in participants without prevalent diabetes and no missing values for confounders at baseline in UK Biobank (n = 350,217)

| **Males** | | | | **Females** | | | |
| --- | --- | --- | --- | --- | --- | --- | --- |
| **HbA1c (mmol/mol)** | | | | | | | |
| **All (n = 160,044)** | | | | **All (n = 190,173)** | | | |
| **Model** | **Regression coefficient^a^** | **95% CI** | ***p-*value** | **Model** | **Regression coefficient^a^** | **95% CI** | ***p-*value** |
| M1 | -0.07 | -0.08, -0.06 | <0.001 | M1 | -0.02 | -0.03, -0.01 | <0.001 |
| **Age group** |  |  |  | **Age group** |  |  |  |
| **<50 years** |  |  |  | **<50 years** |  |  |  |
| M1 | -0.11 | -0.13, -0.08 | <0.001 | M1 | -0.06 | -0.08, -0.04 | <0.001 |
| **50-59 years** |  |  |  | **50-59 years** |  |  |  |
| M1 | -0.08 | -1.00, -0.06 | <0.001 | M1 | -0.06 | -0.07, -0.05 | <0.001 |
| **60-64 years** |  |  |  | **60-64 years** |  |  |  |
| M1 | -0.08 | -0.10, -0.06 | <0.001 | M1 | 0.01 | -0.01, 0.02 | 0.39 |
| **≥65 years** |  |  |  | **≥65 years** |  |  |  |
| M1 | -0.06 | -0.08, -0.04 | <0.001 | M1 | -0.01 | -0.02, 0.01 | 0.55 |
| **Glucose (mmol/l)** | | | | | | | |
| **Model** | **Regression coefficient^a^** | **95% CI** | ***p-*value** | **Model** | **Regression coefficient^a^** | **95% CI** | ***p-*value** |
| M1 | -0.16 | -0.22, -0.11 | <0.001 | M1 | 0.04 | -0.01, 0.08 | 0.09 |
| **Age group** |  |  |  | **Age group** |  |  |  |
| **<50 years** |  |  |  | **<50 years** |  |  |  |
| M1 | -0.23 | -0.37, -0.09 | <0.001 | M1 | -0.01 | -0.10, 0.08 | 0.84 |
| **50-59 years** |  |  |  | **50-59 years** |  |  |  |
| M1 | -0.21 | -0.31, -0.10 | <0.001 | M1 | -0.09 | -0.16, -0.02 | 0.02 |
| **60-64 years** |  |  |  | **60-64 years** |  |  |  |
| M1 | -0.21 | -0.32, 0.09 | <0.001 | M1 | 0.06 | -0.02, 0.14 | 0.13 |
| **≥65 years** |  |  |  | **≥65 years** |  |  |  |
| M1 | -0.09 | -0.20, -0.03 | 0.16 | M1 | 0.10 | 0.01, 0.19 | 0.03 |

^a^ Linear regression models. Model 1 (M1) is adjusted for age, years of education, Townsend index of deprivation, number of long-term conditions, and BMI, leisure-time physical activity, smoking status, alcohol drinking status, and fruit and vegetable intake.

**Table S20.** Odds ratios between glycaemic measures and probable sarcopenia in participants without prevalent diabetes and no missing values for confounders at baseline in UK Biobank (n = 350,217)

| **Males** | | | **Females** | | |
| --- | --- | --- | --- | --- | --- |
| **HbA1c (mmol/mol)** | | | | | |
| **Model** | **OR (95% CI)** | ***p-*value** | **Model** | **OR (95% CI)** | ***p-*value** |
| M1 | 1.02 (1.01, 1.02) | <0.001 | M1 | 1.01 (1.00, 1.01) | 0.03 |
| **Age group** |  |  | **Age group** |  |  |
| **<50 years** |  |  | **<50 years** |  |  |
| M1 | 1.03 (1.01, 1.05) | 0.003 | M1 | 1.03 (1.01, 1.05) | 0.002 |
| **50-59 years** |  |  | **50-59 years** |  |  |
| M1 | 1.02 (1.01, 1.03) | 0.002 | M1 | 1.02 (1.01, 1.03) | 0.002 |
| **60-64 years** |  |  | **60-64 years** |  |  |
| M1 | 1.01 (1.00, 1.03) | 0.05 | M1 | 0.99 (0.98, 1.00) | 0.12 |
| **≥65 years** |  |  | **≥65 years** |  |  |
| M1 | 1.01 (1.00, 1.03) | 0.02 | M1 | 1.01 (1.00, 1.02) | 0.15 |
| **Glucose (mmol/l)** | | | | | |
| **Model** | **OR (95% CI)** | ***p-*value** | **Model** | **OR (95% CI)** | ***p-*value** |
| M1 | 1.05 (1.01, 1.10) | 0.01 | M1 | 1.00 (0.97, 1.04) | 0.78 |
| **Age group** |  |  | **Age group** |  |  |
| **<50 years** |  |  | **<50 years** |  |  |
| M1 | 1.17 (1.06, 1.29) | 0.002 | M1 | 0.99 (0.88, 1.11) | 0.85 |
| **50-59 years** |  |  | **50-59 years** |  |  |
| M1 | 1.10 (1.02, 1.18) | 0.01 | M1 | 1.05 (0.99, 1.11) | 0.12 |
| **60-64 years** |  |  | **60-64 years** |  |  |
| M1 | 1.05 (0.98, 1.13) | 0.18 | M1 | 1.02 (0.97, 1.08) | 0.49 |
| **≥65 years** |  |  | **≥65 years** |  |  |
| M1 | 0.98 (0.92, 1.04) | 0.46 | M1 | 0.97 (0.92, 1.03) | 0.30 |

Model 1 (M1) is adjusted for age, years of education, Townsend index of deprivation, number of long-term conditions, and BMI, leisure-time physical activity, smoking status, alcohol drinking status, and fruit and vegetable intake.

**Table S21.** Associations between glycaemic measures and patterns of grip strength change in participants without prevalent diabetes and no missing values for confounders at baseline in UK Biobank (n = 34,790)

| **All** | | | | **Males** | | | | **Females** | | | |
| --- | --- | --- | --- | --- | --- | --- | --- | --- | --- | --- | --- |
| **n = 34,790** | | | | **n = 16,911** | | | | **n = 17,879** | | | |
| **GS pattern^a^** | **HbA1c**  **(mmol/mol)** | **OR**  **(95% CI)^b^** | ***p-*value** | **GS patten^a^** | **HbA1c**  **(mmol/mol)** | **OR**  **(95% CI)^b^** | ***p-*value** | **GS pattern^a^** | **HbA1c**  **(mmol/mol)** | **OR**  **(95% CI)^b^** | ***p-*value** |
| **Decline** |  | 0.99 (0.98, 1.00) | 0.002 | **Decline** |  | 1.00 (0.99, 1.01) | 0.72 | **Decline** |  | 0.98 (0.97, 0.99) | <0.001 |
| **Stable high** |  | 0.98 (0.97, 0.99) | <0.001 | **Stable high** |  | 0.97 (0.96, 0.99) | <0.001 | **Stable high** |  | 0.99 (0.97, 1.00) | 0.13 |
| **Stable low** |  | 1.02 (1.01, 1.03) | 0.004 | **Stable low** |  | 1.03 (1.01, 1.04) | 0.003 | **Stable low** |  | 1.01 (0.99, 1.03) | 0.31 |
| **GS pattern^a^** | **Glucose**  **(mmol/l)** | **OR**  **(95% CI)^b^** | ***p-*value** | **GS pattern^a^** | **Glucose**  **(mmol/l)** | **OR**  **(95% CI)^b^** | ***p-*value** | **GS pattern^a^** | **Glucose**  **(mmol/l)** | **OR**  **(95% CI)^b^** | ***p-*value** |
| **Decline** |  | 0.95 (0.90, 1.00) | 0.03 | **Decline** |  | 0.96 (0.89, 1.02) | 0.18 | **Decline** |  | 0.94 (0.88, 1.01) | 0.07 |
| **Stable high** |  | 1.00 (0.93, 1.07) | 0.92 | **Stable high** |  | 1.00 (0.91, 1.10) | 0.98 | **Stable high** |  | 0.99 (0.89, 1.10) | 0.83 |
| **Stable low** |  | 0.97 (0.90, 1.04) | 0.37 | **Stable low** |  | 1.00 (0.91, 1.11) | 0.95 | **Stable low** |  | 0.93 (0.83, 1.03) | 0.17 |

^a^GS (grip strength) pattern ‘increase/maintained within the normal range’ (reference). ^b^Multinomial regression models.

Model is adjusted for age, years of education, Townsend index of deprivation, number of long-term conditions, and BMI, leisure-time physical activity, smoking status, alcohol drinking status, and fruit and vegetable intake.


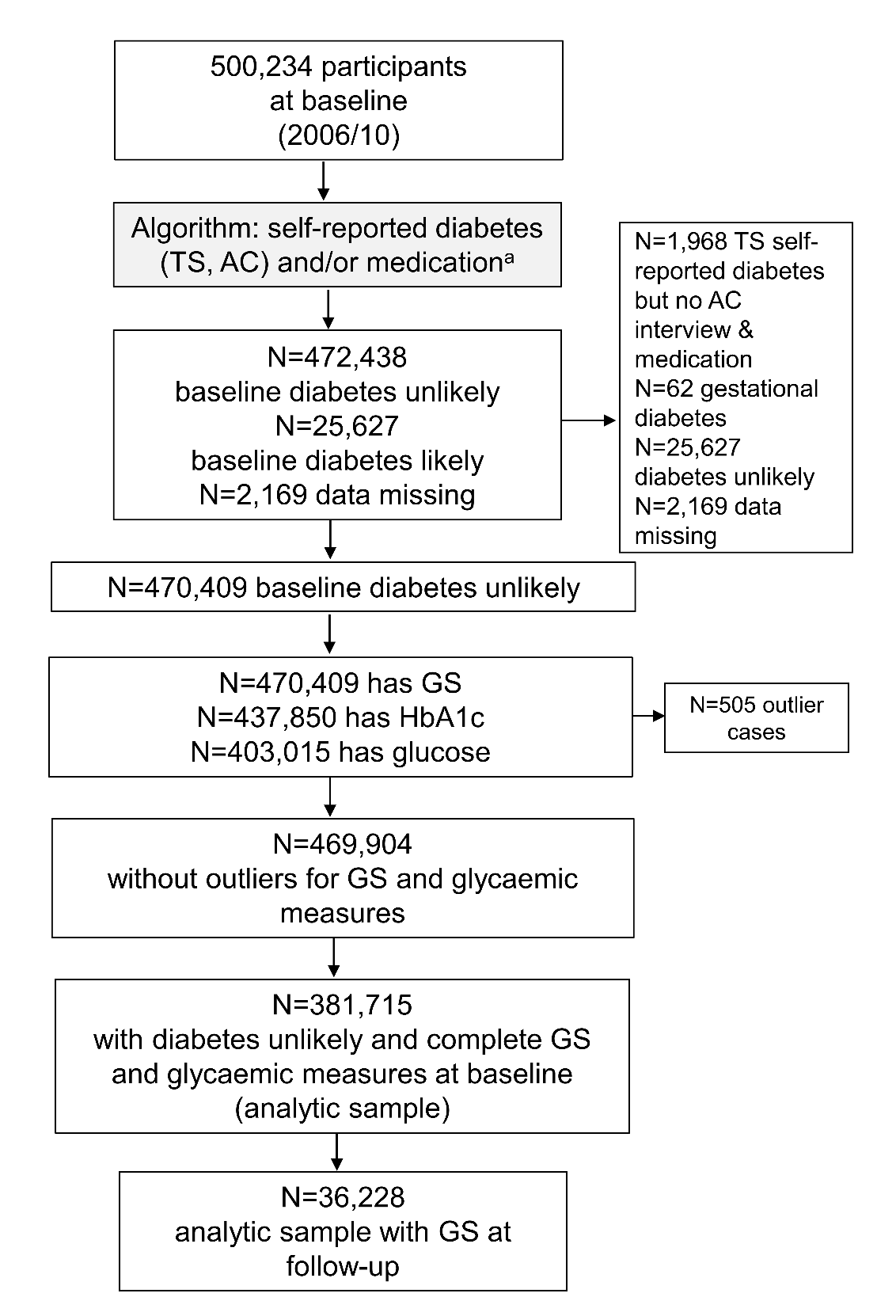


**Figure S1.** Flow chart of participants with ‘diabetes unlikely’ in UK Biobank.

^a^Eastwood et al [25]algorithm for ‘diabetes unlikely’ ascertainment.

AC, Assessment Centre; GS, grip strength; TS, touchscreen questionnaire.
